# Annexin A6 membrane repair protein protects against amyloid-induced dystrophic neurites and tau phosphorylation in Alzheimer’s disease model mice

**DOI:** 10.1007/s00401-025-02888-1

**Published:** 2025-05-24

**Authors:** Katherine R. Sadleir, Karen P. Gomez, Abigail E. Edwards, Armana J. Patel, Makenna L. Ley, Ammaarah W. Khatri, Joanna Guo, Shreya Mahesh, Elyse A. Watkins, Jelena Popovic, Devi Krishna Priya Karunakaran, Dmitry Prokopenko, Rudolph E. Tanzi, Bernabe Bustos, Steven J. Lubbe, Alexis R. Demonbruen, Elizabeth M. McNally, Robert Vassar

**Affiliations:** 1 Department of Neurology, Feinberg School of Medicine, Northwestern University, Chicago, IL 60611 USA; 2 Department of Neurology, Genetics and Aging Research Unit and the McCance Center for Brain Health, Massachusetts General Hospital and Harvard Medical School, Charlestown, MA USA; 3 Simpson Querrey Center for Neurogenetics, Feinberg School of Medicine, Northwestern University, Chicago, IL USA; 4 Department of Pharmacology, Feinberg School of Medicine, Northwestern University, Chicago, IL USA; 5 Center for Genetic Medicine, Feinberg School of Medicine, Northwestern University, Chicago, IL USA; 6 Mesulam Center for Cognitive Neurology and Alzheimer’s Disease, Feinberg School of Medicine, Northwestern University, Chicago, IL USA

**Keywords:** Amyloid pathology, Tau phosphorylation, Membrane repair, Alzheimer’s disease, Calcium dysregulation, Dystrophic neurites

## Abstract

In Alzheimer’s disease, accumulation of amyloid-β (Aβ) peptide is thought to cause formation of neurofibrillary tangles composed of hyperphosphorylated tau protein, which correlates with neuronal loss and cognitive impairment, but the mechanism linking Aβ and tau pathologies is unknown. Dystrophic neurites, which surround Aβ plaques and accumulate phosphorylated tau and other proteins, may play a role in seeding and spreading of pathologic tau. Here, we investigate the novel hypothesis that improved membrane repair capacity decreases dystrophic neurite formation by protecting axons from Aβ-induced membrane damage. Using a ratiometric calcium sensor and a FRET-based calpain cleavage sensor, we demonstrate that dystrophic neurites in 5XFAD mice have elevated resting calcium levels and calpain activity because of putative membrane damage. Annexin A6, a plasma membrane repair in muscle and neurons, is present at plasma membrane of neurons and dystrophic neurites in murine and human brains. Overexpression of annexin A6 in brains of 5XFAD mice decreased size and quantity of dystrophic neurites and accumulation of phospho-tau181, an early biomarker of amyloid pathology. Phospho-tau231, another early amyloid biomarker, and phosphorylated of tau kinases, c-jun N-terminal kinase (JNK) and Calmodulin Kinase II (CaMKII) accumulate in dystrophic neurites in the brains of amyloid pathology mice and humans with AD, suggesting that dystrophic neurites are sites of active tau phosphorylation. Overexpression of dominant-negative annexin A6 in 5XFAD mice increased dystrophic neurites and phospho-tau181. Intracerebral injection of recombinant annexin A6 in 5XFAD and APP-NLGF knock-in mice resulted in localization of recombinant A6 to membranes of dystrophic neurites, suggesting therapeutic potential of recombinant annexin A6 for AD. In conclusion, dystrophic neurites have Aβ-induced membrane damage characterized by calcium elevation, calpain activation, and accumulation of tau kinases and phosphorylated tau. Overexpression of annexin A6 reduces dystrophic neurites and phospho-tau accumulation, suggesting that annexin A6-mediated membrane repair may represent a novel therapeutic approach for AD.

**Supplementary Information:**

The online version contains supplementary material available at 10.1007/s00401-025-02888-1.

## Introduction

Alzheimer’s disease (AD) is the most common cause of dementia affecting 6.7 million people in the US [1]. While therapeutic agents targeting AD pathologies, β-amyloid (Aβ) plaques and tau neurofibrillary tangles (NFTs), are in development and anti-Aβ monoclonal antibodies have been given FDA approval (*e.g.*, aducanumab [2], lecanemab, and donanemab) [84], there is still need to develop disease-modifying therapeutics targeting other pathologic mechanisms of AD to be used separately or in combination. Anti-Aβ antibodies slow but do not halt or reverse AD progression, while benefiting only early AD and having potentially serious side effects. Therefore, discovering novel disease-modifying drugs that can benefit all stages of AD with minimal adverse effects is a high priority in the field.

Accumulation of cerebral Aβ precedes the formation of tau tangles by at least 2 decades in AD [5, 18, 49, 50, 57–59, 69, 80, 87, 88]. Moreover, amyloid-positivity is associated with cognitive decline and tau accumulation [15, 49, 83, 87]. This evidence suggests that toxic Aβ species (*e.g.*, oligomers, protofibrils, and fibrils) trigger pathologic tau (tau hyperphosphorylation, fragmentation, and NFTs), which is associated with synapse and neuron loss (reviewed in [66] Although the mechanism linking Aβ and tau in AD is a profound mystery, disrupting the link between amyloid and tau pathologies, especially before cognitive impairment, may represent an effective therapeutic strategy for preventing AD.

Dystrophic neurites (DNs) are swollen neuronal processes, mostly axons [77, 98, 106], which form near amyloid plaques in the AD brain, often creating a halo that surrounds and is in contact with the plaque (reviewed in [111]). DNs accumulate proteins involved in AD, such as amyloid precursor protein (APP) [4, 22, 24, 62], β-site APP cleaving enzyme 1 (BACE1) [64, 98, 121], and abnormally phosphorylated tau (reviewed in [32]). We and others hypothesize that accumulation of phosphorylated, truncated, and aggregated tau in plaque-associated DNs plays a key role in the spread of pathologic tau throughout the brain [10, 53, 73, 119] and may link Aβ and tau pathologies. It has long been observed that DNs around cored plaques stain with antibodies to a variety of different pathological phospho-tau epitopes, such as AT8, and antibodies recognizing paired helical filaments (PHF) [32]. Increased cerebral spinal fluid (CSF) and plasma levels of tau phospho-isoforms, such as p-tau181, p-tau217, and p-tau231, accurately predict amyloid-positivity by positron emission tomography (PET) and are robust biomarkers of AD status and severity [19, 45, 60, 65, 107], further supporting a link between Aβ and tau. A direct relationship exists between the amount of neuritic dystrophy around Aβ plaques and the spread of pathologic tau from one hemisphere to the other in amyloid mouse models after injection of tau seeds isolated from human AD brain [47, 53, 72, 97]. Mouse models, such as 5XFAD, with many neuritic plaques have more extensive tau spreading than those with fewer or more diffuse plaques, such as APP-NLF knock-in mice [53]. Additionally, mutations in *TREM2* result in increased DNs around plaque and promote pathologic tau spreading in mouse models [72]. Moreover, humans with no cognitive impairment, but levels of amyloid plaques similar to those of AD patients at autopsy (termed asymptomatic AD), had significantly less phospho-tau accumulation around plaques, implicating DN-associated phospho-tau in cognitive decline [63]. Interestingly, tau isolated from these asymptomatic AD brains had aggregation states and seeding properties that were comparable to tau isolated from amyloid plaque-free control brains and lower than tau isolated from AD brains [63]. Together, these data suggest that preventing the formation of DNs should slow the development of tau pathology and benefit cognition.

Although an association exists between Aβ plaques and DNs, the mechanism of DN formation is unclear. One hypothesis is that DNs result from axonal membrane damage caused by contact with Aβ in plaques. Aβ peptides, especially longer isoforms, like 42 amino-acid Aβ42, are very hydrophobic and may injure cell membranes. Indeed, evidence suggests that neuronal membrane damage caused by Aβ could be involved in the etiology of AD [33, 37, 51]. One of the most direct effects of plasma membrane damage is increased intracellular calcium. The calcium hypothesis of AD posits that dysregulation of calcium homeostasis is the point of convergence of many risk factors and molecular mechanisms that lead to development of AD and its associated neurodegeneration [3, 67]. Basal intracellular calcium levels are abnormally elevated in neuronal soma and DNs near amyloid plaques in *APP* transgenic mice, such that an increasing calcium concentration gradient is observed approaching the plaque, as opposed to regions distant from plaques in which calcium levels appear normal [68]. Increased intracellular calcium can affect activity of tau kinases and phosphatases and activate calpain cleavage of tau [38, 91, 103], which can create toxic tau fragments [91] and affect tau phosphorylation and aggregation states (reviewed in [11]). Overexpression of the endogenous calpain inhibitor calpastatin and pharmacological inhibition of calpain have been shown to ameliorate the phenotypes of amyloid and tau mouse models [74, 82].

Since membrane damage is a common threat to cellular viability, mechanisms have evolved to repair and reseal the plasma membrane. This function is probably most important in tissues with long-lived cells that suffer mechanical stress, such as skeletal and cardiac muscle, and have limited replacement potential, such as neurons [29]. The annexin family of proteins exhibit broad tissue distribution and function in membrane and actin remodeling, immune cell modulation, and wound repair [48]. Annexin A6 (A6) plays a key role in membrane resealing in skeletal muscle, heart, and neurons, and recombinant A6 shows therapeutic potential in these systems [30, 31]. Like other annexins, A6 binds negatively charged phospholipids, such as phosphatidylserine, phosphatidic acid, and phosphoinositides, in the presence of calcium [44]. Additionally, A6 is unique among the annexins in that the gene originated from a genomic duplication resulting in eight calcium-binding repeats rather than four (Fig. 3A). Upon acute membrane injury, A6 binds calcium and initiates the formation of a repair cap protein complex at the site of damage (Fig. 3B) [31]. After calcium binding, annexin A6 [34, 99, 116] then recruits other proteins in the repair complex, annexins A1 and A2 [34, 90], and the shoulder proteins BIN1 [40, 110], dysferlin [39, 54], and the EHD family proteins [12, 13, 43], which are all expressed in brain. *BIN1* is a strong GWAS AD risk factor [26, 27, 70, 102], suggesting that the membrane repair function of BIN1 may be compromised in AD. A truncation mutant resulting in only the N-terminal 32 amino acids of annexin A6, p.N32* [108], and a missense mutation that is deficient in calcium binding, p.E233 A [30], both exert a strong dominant-negative effect on repair cap assembly, supporting the key role of annexin A6 in membrane repair.

Recently, we have demonstrated that acute plasma membrane laser injury of annexin A6-GFP knock-in mouse primary neurons results in rapid recruitment of A6-GFP at the site of injury [29], similar to the well-established laser-induced muscle injury paradigm [28, 30, 31, 108]. In skeletal and heart muscle cells, exogenous recombinant annexin A6 (rA6) localizes rapidly to the site of laser injury and enhances membrane repair, suggesting that rA6 may offer a novel therapeutic approach [29, 30]. Similarly, we have shown that recombinant tdTomato-tagged annexin A6 added to the media colocalized with genomically expressed annexin A6-GFP at the site of laser injury site in both neuronal soma and neurites of primary neurons [29]. These results suggest that, like in muscle, either annexin A6 gene therapy or exogenous rA6 administration could be used therapeutically to mediate neuronal membrane repair in the brain. These *in vitro* results are intriguing, but do not directly address whether annexin A6 may have a role in membrane repair in AD.

Here, we investigate the link between annexin A6, Aβ-induced membrane damage, calcium and calpain dysregulation, DN formation, tau kinase activation, and subsequent phospho-tau accumulation. We demonstrate that DNs have raised resting calcium, elevated calpain activity, increased phosphorylated tau kinases, and accumulation of the amyloid biomarkers p-tau181 and p-tau231, together suggesting that DNs promote an environment of pathological tau phosphorylation and cleavage. We show that the membrane repair protein annexin A6 is expressed in neurons of human and murine brains, and overexpression of annexin A6 in the 5XFAD amyloid mouse model reduces DNs and p-tau181, while dominant-negative annexin A6 increases DNs and p-tau181. Promisingly, recombinant annexin A6 injected into 5XFAD and APP-NLGF knock-in mouse brains shows the ability to bind to the membranes of DNs around amyloid plaques suggesting that, like in muscular dystrophy, annexin A6 protein could be investigated as a therapeutic. These data support the further investigation of Aβ-induced membrane damage as an etiological mechanism of DN formation and AD pathogenesis, and the potential therapeutic use of annexin A6 to mediate membrane repair that would disrupt the link between Aβ and tau pathologies for the prevention of AD.

## Materials and methods

### Mice

Two lines of 5XFAD mice were used in this study. 5XFAD B6/SJL F1 hybrid on mice was bred in-house by crossing 5XFAD transgenic males to B6/SJL F1 hybrid females and genotyped as described [86]. 5XFAD mice on a C57BL/6 J background were maintained by crossing 5XFAD transgenic males to C57BL/6 J females and genotyped using 5XFAD probes at Transnetyx. APP-NLGF mice on a C57BL/6 J background were obtained from Riken (RBRC06344) and the line was maintained by crossing homozygotes. Genotyping was performed using Riken probes through Transnetyx (Memphis, TN, USA). To obtain 5XFAD;Tau^-/-^ tissue, 5XFAD B6/SJL F1 hybrid males were crossed to Tau^-/-^ females from Jackson Laboratories (strain number 007251). The resulting 5XFAD;Tau^+/-^ males were crossed to Tau^-/-^ females to obtain 5XFAD;Tau^-/-^ animals that were harvested at 9 months of age. Genotyping for tau was performed as described on Jackson Laboratories website and in original reference [25]. Mice were sacrificed by a lethal dose of ketamine/xylazine, followed by transcardial perfusion with 10 ml ice cold 1xPBS containing protease and phosphatase inhibitors. One hemibrain was dissected into hippocampus and cortex which were snap-frozen separately. The other hemibrain was drop fixed in 10% buffered formalin overnight at 4 °C, transferred to 20% w/v sucrose in 1xPBS for 24 hours, and then stored in 30% w/v sucrose in 1xPBS with azide. All animal work was done with the approval of the Northwestern University IACUC, assurance number A3283-01. The number and sex of animals used per experiment is described in detail in the specific methods section of the experiment.

### AAV PHP.eB and retro-orbital injections for calcium imaging and calpain sensor

Synapsin-GCaMP6s-ER/K linker-TdTomato (VB230218-1013vfu) and calpain sensor synapsin-Cerulean-PLFAAR-Venus (VB231102-1456esn) were generated and packaged into AAV PHP.eB by Vectorbuilder. For retro-orbital injections, mice were anesthetized in 2.5% isoflurane and 1% oxygen, and then received 100 ul of virus into the retro-orbital sinus, using a 30 gauge needle.

For calcium imaging, five 5XFAD female mice and two non-Tg littermates received 5.5x10^11^ viral genomes of syn-GCaMP6s-ERK-TdT at 5–6 months of age and were collected for live imaging 4 weeks later at 6–7 months of age. Twenty-four hours prior to live imaging, mice received intraperitoneal injection of 100 ul of 5 mg/ml MeX04 (HelloBio) in 15% DMSO, 45% propylene glycol, and 40% PBS pH 7.5 24 hours before harvest. Mice were anesthetized with ketamine/xylazine then transcardially perfused with superchilled oxygenated sucrose artificial cerebrospinal fluid (ACSF). One hemibrain was sliced on a vibratome in chilled oxygenated sucrose ACSF solution to 300μm coronal sections. Sections were warmed to 32 °C and equilibrated into oxygenated ACSF, and then cooled to room temperature for imaging. Slices were imaged at room temperature in ASCF for no more than 30 min. Images were collected with a 25x dipping lens on a Nikon A1R Multiphoton with Chameleon Vision titanium sapphire laser tuned to 920 nm for GCaMP6s/TdTomato and 760 nm for MethoxyX04. Images were collected every 2 μm for 50–70 μm z-stacks. In FIJI, maximum projections were generated from the stacks, Regions of interest (ROIs) were drawn around cell bodies and dystrophic neurites, and the ratio of GCaMP6s intensity to TdTomato intensity calculated for each ROI. For each mouse (*n* = 5), 81–131 cell bodies and 407–673 dystrophic neurites from 6 to 9 Z stacks from 3 individual slices were used for quantification. Data are presented as the average for each mouse.

For calpain activity measurement, 5XFAD and non-Tg littermates (*n* = 3) received 1 x 10^12^ viral genomes of Cerulean-PLFAAR-Venus (CFP–PLFAAR–YFP) at 7.5–9.5 months of age and were harvested 5 weeks later for immunofluorescence and biochemistry, as described above. Formalin-fixed brains were sectioned at 30 μm, and incubated with 1:15,000 of 1 mg/ml Thiazine red to label plaques. Imaging was performed on Nikon A1R confocal with a 20x NA 0.75 lens, and images captured in NIS-Elements software (Nikon). The CFP, YFP, and FRET (YFP emission wavelength with CFP excitation) intensities were quantified from single confocal images in FIJI. Three-to-four random cortical fields were used per mouse (*n* = 2) containing 2–11 neuronal soma and 9–64 dystrophic neurites per image for a total of 46 cell bodies and 245 dystrophic neurites.

### AAV8 and P0 injections

Synapsin-A6GFP (VB200511-1403pbc), synapsin GFP (VB200603-1246qqd), and synapsin-A6 N32GFP (VB210122-1156evb) AAV plasmids were generated and packaged in AAV 8 serotype by VectorBuilder (Chicago, IL USA). Genomic titer was determined by quantitative PCR. 5XFAD transgene positive males were crossed to SJL/B6 hybrid females in timed matings to generate transgene negative and positive littermates. On P0, each pup in a litter (4 litters per virus, 22–28 pups) was cryo-anesthetized and injected with 2 µl/hemisphere containing 2 × 10^11^ viral genomes of Syn-A6GFP, or 2 × 10^10^ viral genomes of Syn-GFP, to compensate for the higher expression of GFP alone. For syn-A6 N32-GFP, 2 × 10^10^ viral genomes/hemisphere were injected, due to known toxicity of the protein in muscle [108]. All injections were done using a using a 10 µl Hamilton syringe with a 30 gauge replaceable needle, as described in [71]. Pups were returned to the mother and aged to 4.5 months. All 5XFAD transgene positive males were used for analysis (4–6 per AAV).

### Recombinant A6-HIS and A6-GFP and stereotaxic injection

Recombinant annexin A6-HIS tagged protein was generated by Evotec SE (Hamburg, Germany) contract research service for protein purification using *E*. *coli* as described in [29]. The AbVec2.0 vector was used to express A6-GFP intracellularly in HEK293 suspension cells with a 6X-Histidine tag to allow for purification via Ni-NTA chromatography. HEK293 suspension cells were grown with the FreeStyle Expression System (Gibco) and transfections performed using polyethylenimine and OptiPRO SFM (Gibco). Cells were pelleted and lysed in mild detergent M-PER (Thermofisher) containing protease inhibitor and benzonase nuclease. Protein from the lysates was purified via their His-tag using cOmplete Ni-NTA resin (Roche) and dialyzed against PBS, and then quantified on a NanoDrop (Thermofisher)

For stereotaxic injection of recombinant annexin A6-HIS and A6-GFP, 5XFAD and APP-NLGF mice were anesthetized with isoflurane (1.5–2.5%) and 1% oxygen in a stereotaxic apparatus. The lateral ventricle (B-L −0.5; M-L+/–1; D-V −2.5) or the dentate gyrus (B-L −2.5; M-L +/−2.0; D-V −2.2) and overlying cortex (B-L −2.5; M-L +/−2.0; D-V −2.0) were targeted. For A6-HIS, 5XFAD (n=5 males, 5–10 months old) or NLGF mice (*n* = 4, 2 males, 2 females, 9–11 months old) received 0.67 mg/kg into the hippocampus and cortex, or 6.6 mg/kg into the ventricle. Both injection targets produced similar subcellular localization of protein with different anatomic distribution, For A6-GFP, 5XFAD (*n* = 2 males, 11 months old) or NLGF mice (*n* = 3, 2 male, 1 female, 9–10 months old) received 0.45–0.9 mg/kg into the hippocampus and overlying cortex. Animals were sacrificed and tissue harvested 3–5 hours later, as described, and tissue sectioned and stained for imaging. 3–5 hours was chosen as a time frame that allowed protein to diffuse and bind to targets, but before it was degraded appreciably.

### Immunofluorescence and image quantification

10% formalin-fixed hemibrains were sectioned at 30 μm on a freezing sliding microtome and collected in cryopreserve (30% w/v sucrose, 30% ethylene glycol in 1X PBS) then stored at – 20 °C. If required, antigen retrieval was performed for 45 min at 80 °C in 0.1M sodium citrate, in TBS, and then allowed to cool for 15 min before rinsing well and proceeding with staining. Sections were blocked in 5% normal donkey serum in TBS with 0.1% Triton-X 100 (TBS-T) and then primary antibodies added in 1% BSA in TBS-T at 4 °C overnight. Primary antibody concentrations can be found in Table 1. Following washes, sections were incubated for 2 hours at room temperature with 1:750 dilutions of Alexa Fluor 405, 488, 568, or 647 conjugated donkey anti-mouse, goat, chicken, rat, or rabbit secondary antibodies, as appropriate, along with the following stains: 300 nM DAPI, and 1:15,000 dilution of 1 mg/ml Thiazine Red (ThR) or MethoxyX04 (MeX) (HelloBio). All secondary antibodies were obtained from ThermoFisher Scientific, except donkey anti-chicken 405, 488, and 647 were from Jackson Immunologicals (West Grove, PA, USA) and donkey anti-rat 568 from Abcam (Waltham, MA USA). Sections were mounted with Prolong Gold (Molecular Probes). For protein localization, images were captured from 2–3 sections per mouse, 2–3 mice per sex and genotype, comprising male and female mice between 6 and 9 months of age, on a Nikon A1R confocal microscope using 60x objective.

**Table 1:** List of primary antibodies used for immunoblotting and immunofluorescence

Immunoblot antibodies				
Protein	Company	cat #	conc	Species
Annexin A6	Abcam	Ab199422	1:5000	Rabbit
GFP	Abcam	Ab5450	1:1000	Goat
APP 6E10	Biolegend	#803001	1:1000	Mouse
β-tubulin	Gift of Nick Kannan	TuJ1 clone	1:10:000	Mouse
FLAG	ProteinTech	80010-1-RR	1:1000	Rabbit

Immunofluorescence antibodies					
Protein	Company	cat #	conc	Species	Antigen retrieval
Iba1	Novus	NB100-1028	1:300	Goat	
Annexin A6	Abcam	Ab199422	1:300	Rabbit	AR needed
GFAP	Abcam	Ab4674	1:2000	Chicken	
BACE1	Abcam	Ab108394	1:300	Rabbit	
BACE1	Vassar Lab	clone 3D5	1:300	Mouse	AR needed
hAPP N-term	Gift of Virginia Lee	karen	1:2000	Goat	
APP C-term Y188	Abcam	Ab32136	1:2000	Rabbit	
Aβ42	Invitrogen	#700254	1:1000	Rabbit	
NeuN	Millipore	Abn91	1:1000	Chicken	
NeuN	Millipore	Mab377	1:200	Mouse	
Aβ	Gift of Elan	3D6	1:1000	Mouse	
LAMP1	DSHB	1D4B clone	1:1000	Rat	Not AR compatible
p-tau 181	Cell signaling	#12885	1:300	Rabbit	
p-tau 231	Abcam	ab151559	1:500	Rabbit	
p-JNK	Cell signaling	#9255	1:500	Mouse	
p-CaMKII	Abcam	ab5683	1:300	Rabbit	
tau	Gift of Nick Kannan	tau5	1:300	Mouse	
anti-HIS	Cell signaling	#12698	1:500	Rabbit	
Annexin A1	Abcam	ab214486	1:300	Rabbit	AR needed
Annexin A2	Abcam	ab189473	1:1000	Rabbit	AR needed
GFP	Abcam	Ab5450	1:5000	Goat	

For quantification of LAMP1 and p-tau181 in dystrophic neurites, and Iba1 and GFAP quantification around plaques, three sections between Bregma −1.58 mm and Bregma −2.54 mm were stained and imaged per mouse. Images were acquired on a Nikon Ti2 Eclipse widefield microscope with a 10x objective, using the NIS-Elements software high content method to capture and tile whole sections. All image acquisition settings were maintained the same between treatment groups and genotypes. For image analysis, ROIs were drawn in cortex and hippocampus. Using the General Analysis tool, thresholding was set to distinguish Aβ42, LAMP1, MethoxyX04, Thiazine Red, and p-tau181 positive regions, and then, the percent area covered by each stain from the hippocampal or cortical ROI was calculated. Using the same sections, thresholding in the General Analysis tool was used to define sub-ROIs that correspond to individual plaques having both Aβ42 and LAMP1 or p-tau181 and Thiazine red positive pixels within cortical and hippocampal ROIs. The General Analysis tool was used to measure area covered by Aβ42 and by LAMP1, or p-tau181 and ThR or p-tau181 and MeX in a given sub-ROI (plaque), and the ratio between LAMP1:Aβ42, p-tau181:ThR or p-tau181:MeX was calculated in Excel. 615–1393 plaques were analyzed per mouse in cortex and 210–527 plaques in hippocampus. Four-to-five male mice per genotype/treatment group were analyzed, using three sections per mouse.

For measurement of area of GFP-positive (GFP+) dystrophic neurites, three sections from each mouse (5 A6-GFP injected and 5 GFP injected) were imaged on a Nikon A1R confocal with a 60x objective. The section of cortex directly above hippocampus to the top of layer 5 of the cortex was imaged, and a tiled imaged of multiple fields (8 × 4) was generated to create a single image 1474.36 by 753.23 microns. Within this region, Nikon NIS-Elements software was used to threshold GFP+ structures, excluding cell bodies based on size, and the size of each GFP+ structure was measured. 1761–4941 GFP+ dystrophic neurites were measured per mouse.

### Human tissue

30 μm floating sections of hippocampal tissue from 3 individuals with autopsy confirmed AD pathology (1 male, 2 female, ADNC high—A3, B3, C3), and 3 non-cognitively impaired age-matched controls (2 male, 1 female) were obtained from the brain bank of the Mesulam Center for Cognitive Neurology and Alzheimer’s Disease at Northwestern University. Immunofluorescence was performed as described for mice, except that sections were blocked in 10% normal donkey serum, with 2% BSA in TBS with 0.1% TBS-T and primary antibodies added over night in 2% normal donkey serum with 2% BSA in TBS-T at 4 °C. As a final step before mounting, sections were incubated 7 min in TrueBlack (#23014 Biotium) diluted 1:40 in TBS.

### HEK293 and SH-SY5Y cell culture (supplemental figures)

HEK293 cells were obtained from ATCC and cultured in DMEM with glucose, 10% fetal bovine serum, and 1% penicillin/streptomycin all from ThermoFisher scientific. Calpain 1 small subunit expression construct was obtained from Origene (RC212322) and calpain 1 large subunit was obtained from Addgene (60941). Both plasmids, along with UBC-CFP–PLFAAR–YFP from VectorBuilder, were prepared with Machery Nagel MuceloBond Xtra maxi kits and transfections performed with Lipofectamine 2000 (Invitrogen). After 24 h, cells were lysed in RIPA buffer and sonicated, and protein concentration was quantified using BCA Assay (Pierce). 10 μg of protein was boiled 10 min in 1x LDS sample loading buffer, and immunoblotting performed as described below. For FRET imaging, cells were plated on coverslips, and then, 24 h after transfection were fixed in 4% paraformaldehyde, mounted on slides, and imaged, as described previously for FRET. SH-SY5Y cells were obtained from ATCC and cultured in DMEM with glucose, 10% fetal bovine serum, and 1% penicillin/streptomycin, all from ThermoFisher scientific. For imaging, cells were plated on coverslips coated with laminin in media containing retinoic acid to induce differentiation. GCaMPs-TdTomato was transfected into SH-SY5Y cells using Lipofectamine 2000. Cells were imaged on 25x dipping lens on a Nikon A1R Multiphoton with Chameleon Vision titanium sapphire laser tuned to 920nm for GCaMP6 s/TdTomato, in Ringer’s with calcium (155 mM NaCl, 4.5 mM KCl, 5 mM HEPES, 10 mM D-Glucose, 1 mM MgCl_2_, and 2 mM CaCl_2_). Images were captured every 5 s for 5 min, with 90 mM KCl in Ringer’s with calcium infused in after 1 min of imaging to depolarize cells. Calcium levels were quantified using FIJI as described above, taking the ratio of GCaMP6 s to TdTomato in each cell.

### Immunoblotting

Snap frozen cortices and hippocampi were homogenized in 1000 μl or 300 μl, respectively, of T-PER Tissue Protein Extraction Reagent (ThermoFisher), supplemented with protease inhibitors (Calbiochem) and Halt Phosphatase Inhibitor Cocktail (Thermo Scientific). Protein concentration was quantified using BCA Assay (Pierce). 20 μg of protein was boiled 10 min in 1X LDS sample loading buffer, and then separated on 4–12% NuPage Bis-Tris Bolt gels (FisherScientific) in MES buffer (FisherScientific). Protein was transferred to nitrocellulose membrane using BioRad Trans_Blot Turbo Transfer System, then stained with 0.1% Ponceau in 5% w/v acetic acid, and imaged. Membrane was rinsed well, blocked, and then probed with the following primary antibodies: anti-APP (6E10, BioLegend 803001, 1:2000), anti-Annexin A6 (Abcam ab 1:5000), anti-β-tubulin (TuJ1, gift of Dr. Nicholas Kanaan, 1:10,000), anti-FLAG (ProteinTech, 80010-1-RR, 1:1000), anti-GFP (Abcam ab5450, 1:1000) followed by HRP-conjugated anti-mouse, anti-rabbit, or anti-goat secondary antibody (Vector Laboratories 1:10,000). 5% milk was used as a blocking agent. Blots were visualized using chemiluminescence (BioRad Clarity), and band intensities measured using a BioRad ChemiDoc Touch Imaging System and then quantified with the ImageLab software (BioRad). Signal intensities were normalized to that of tubulin. Statistical analyses were performed as described below.

### Protein schematics

Protein ribbon diagrams were generated using Swiss-PdbViewer using solved crystal structures of human annexin A6 with phosphorylation mimicking mutant T356D (1M9I), www.rcsb.org.

### Statistics

Student’s two-tailed t test and ANOVA were done using InStat software (GraphPad Software, Inc., San Diego, CA) to compare means of the various genotypes, genders, and treatment groups. * 0.05 > *p* > 0.01 ** 0.01 > *p* > 0.001 *** 0.001 > *p* > 0.0001; Error bars = S.E.M.

## Results

### Dystrophic neurites have elevated resting calcium

**Fig. 1:**
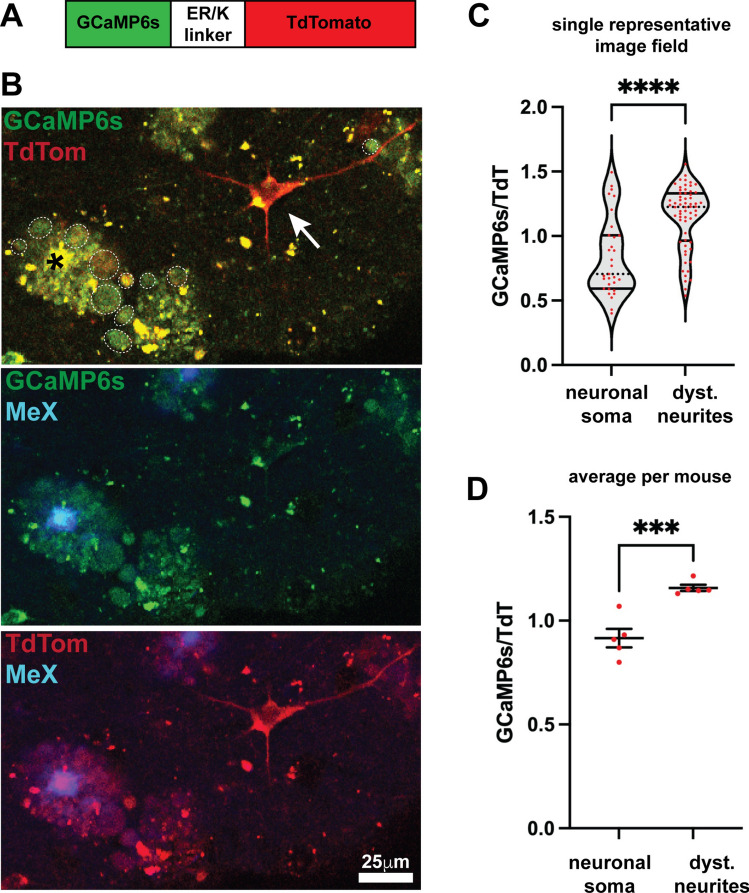
Resting calcium levels are elevated in dystrophic neurites of 5XFAD mice. **A** Schematic of ratiometric calcium sensor GCaMP6s-ERK-TdTomato. **B** 300 μm live slices of 5XFAD (*N* = 5) mice injected with AAV PHP.eB syn-GCaMP6s-ERK-TdT were imaged on a Nikon A1R confocal multiphoton microscope at 25x. Upper panel of B shows an example of a neuronal soma with low GCaMP6s fluorescence (arrow) and dystrophic neurites with higher GCamP6s fluorescence (dotted circles). Lower panels show MethoxyX04 (MeX) marking dense core amyloid plaques and the fluorescence of GCaMP6 s (middle panel) or TdTomato (lower panel) independently. Imaging of brain slices from 5XFAD mice without GCaMP6s-ERK-TdTomato expression indicated that bright signals from plaques and puncta are autofluorescence of live tissue (Suppl. Fig. 1C). **C**, **D** For each z stack (*n* = 6–9 per mouse, from 3 different slices, 5 mice), the ratio of GCaMP6s:TdT was quantified in neuronal soma and dystrophic neurites using FIJI. **C** All neuronal soma and dystrophic neurites from one representative z stack are shown, indicating a significant increase in ratio in dystrophic neurites, but with wide variation (image shown in Sup. Fig 1D). Dotted black line represents median, solid black lines represents quartiles, and red points indicate individual cells or dystrophic neurites. **D** The average GCaMP6s:TdT ratio per mouse (*n* = 6–9 z-stacks per mouse, from 3 different slices, 5 mice) is shown, demonstrating a significantly increased GCaMP6s:TdT ratio, but with decreased variation. Two tailed Students t test, ****p < 0.0001, ***p < 0.001. Red points represent individual mice, with mean and SEM

Since we hypothesized that contact with amyloid plaques disrupts the membrane of nearby axons leading to calcium influx and subsequent disruption of kinases and phosphatases, microtubule-based transport, and ultimately neuronal function, we wanted to assess steady-state resting calcium levels in DNs. To do so, we generated a sensor fusion protein, GCaMP6s-TdT, consisting of the highly sensitive calcium sensor GCaMP6s [16] fused to calcium insensitive TdTomato (TdT), which was included to normalize for sensor protein expression (Fig. 1A). Our calcium sensor allowed us to measure steady-state resting calcium, rather than calcium change over time, as is more commonly done using GCaMP variants alone. We inserted a stiff α-helical 30 residue ER/K linker between GCaMP6s and TdT to increase the distance between the two fluorescent proteins for minimizing FRET interactions [104, 109]. We verified the calcium responsiveness of GCaMP6s-TdT by measuring GCaMP6s:TdT fluorescence ratio during depolarization. SH-SY5Y cells were transfected with the neuron-specific human synapsin-GCaMP6s-ER/K-TdT (hSyn-GCaMP6s-ER/K-TdT) plasmid, differentiated with retinoic acid, and then imaged using multiphoton microscopy while being depolarized with 90 mM KCl. We observed that the ratio of GCaMP6s:TdT fluorescence was increased by about tenfold in depolarized cells compared to non-depolarized cells (Supplemental Fig. 1A, B).

To assess resting calcium levels *in vivo* in DNs, we packaged syn-GCaMP6s-ER/K-TdT into AAV PHP.eB, which crosses the blood–brain barrier when injected intravenously [14, 55]. 6–7 month-old 5XFAD mice were retro-orbitally injected with syn-GCaMP6s-ERK-TdT AAV and brains collected 5 weeks later and sliced for live multiphoton microscopy imaging. To label plaques in live brain slices without relying on antibody staining, mice received a single IP injection of the brain-penetrant small molecule amyloid dye, Methoxy-X04, 1 day before harvest. DNs around dense core plaques labeled with Methoxy-X04 showed more green GCaMP6s on average than neuronal cell bodies (Fig. 1B). The GCaMP6s:TdT fluorescence ratio was quantified for individual DNs and neuronal soma in single 50–70 mm z-stack images (Fig. 1C) and as the average DN and soma ratios per mouse (5 mice, 6–9 DN and soma images each from 3 slices per mouse; Fig. 1D). Overall, DNs showed a significant increase in GCaMP6s:TdT ratio in compared to cell bodies, indicating increased resting calcium levels in DNs. Live multiphoton microscopy imaging of 5XFAD brain slices without syn-GCaMP6s-ER/K-TdT AAV injection demonstrated green- and red-channel autofluorescence in plaque cores and puncta in neuronal soma and surrounding neuropil (Supplemental Fig. 1C). We verified that the formation of DNs is not dependent upon the intraneuronal expression of human APP from the 5XFAD transgene by performing co-staining with a human-specific N-terminal APP antibody [112] and an APP antibody that recognizes both mouse and human APP (rabbit monoclonal Abcam ab32136), or an antibody recognizing BACE1, demonstrating that not all dystrophic neurites contain human APP (Supplemental Fig. 2). Rather, DNs appear to form as a response to amyloid plaques in close proximity.

### Dystrophic neurites have increased calpain activity

**Fig. 2:**
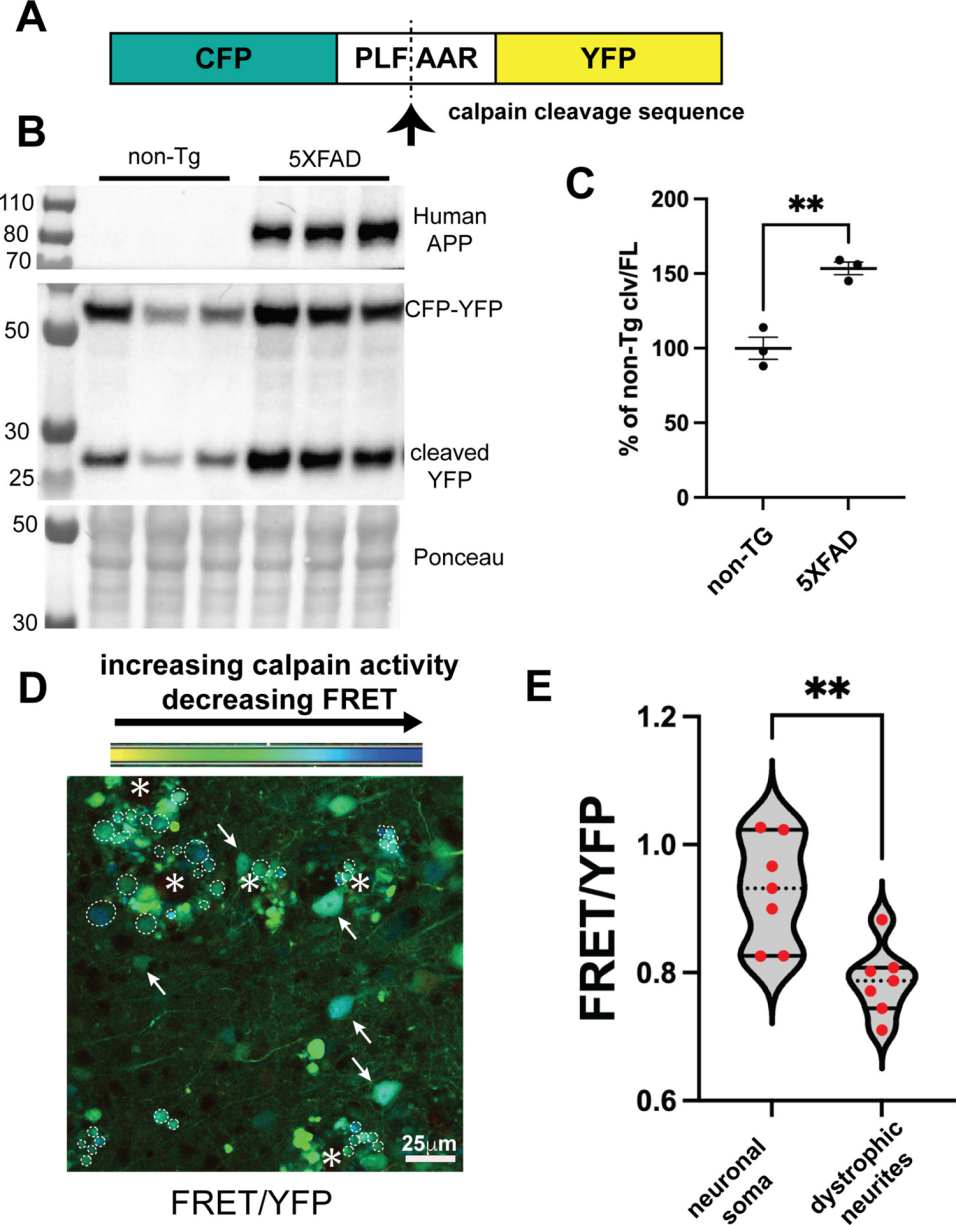
Calpain activity is elevated in dystrophic neurites of 5XFAD mice. **A** Schematic of calpain sensor CFP–PLFAAR–YFP. Arrow indicates calpain cleavage site. **B**, **C** At 6–7 months of age, 5XFAD mice and non-transgenic littermates (n=3 each genotype) received a retro-orbital injection of AAV PHP.eB syn-CFP–PLFAAR–YFP. After 5 weeks of expression, brains were collected and immunoblot analysis performed, which indicated significantly elevated ratio of cleaved to full-length CFP–PLFAAR–YFP in 5XFAD mice compared to non-Tg mice. **D**, **E** Using FIJI, FRET and YFP intensity were measured in neuronal soma (arrows) and dystrophic neurites (dotted circles). **E** The ratio of FRET:YFP was significantly decreased in dystrophic neurites compared to neuronal cell bodies, indicating an increase in calpain activity in dystrophic neurites. For (**E**), 3–4 random cortical fields were used per mouse (*n* = 2) containing 2–11 neuronal soma and 9–64 dystrophic neurites per image for a total of 46 cell bodies and 245 dystrophic neurites. Each red data point represents the ratio in a cortical field. Dotted black line represents median and solid black lines represent quartiles

Since calcium levels control the activity of the protease calpain, which is known to cleave tau potentially leading to the generation of aggregation-prone toxic fragments [11, 91], we also developed a calpain sensor to assess calpain activity in DNs. The calpain sensor construct (CFP–PLFAAR–YFP) consists of cerulean fluorescent protein (CFP) fused to mVenus yellow fluorescent protein (YFP) via a calpain cleavage site linker referred to by its amino-acid sequence, PLFAAR (Fig. 2A) [81]. To test the specificity of our calpain sensor, we expressed it in HEK293 cells with or without co-expression of the calpain 1 large and small subunits. Cleavage of CFP–PLFAAR–YFP was observed only when the calpain subunits were co-expressed, demonstrating specific detection of calpain activity (Supplemental Fig. 3A). A negative control calpain sensor with a GGGGS linker that cannot be cleaved by calpain, CFP-GGGGS-YFP, remained full length following co-expression of the calpain 1 large and small subunits (Supplemental Fig. 3A).

Next, we used our calpain sensor to assess calpain activity *in vivo* in DNs of 5XFAD mice. CFP–PLFAAR–YFP under control of the synapsin promoter (syn-CFP–PLFAAR–YFP) was packaged into AAV PHP.eB and retro-orbitally injected into 7-month-old 5XFAD mice and non-transgenic (non-Tg) littermates that were aged 5 weeks and brains harvested for immunoblot analysis of brain homogenates and FRET in brain sections. By immunoblot, we observed significantly increased cleavage of CFP–PLFAAR–YFP in 5XFAD compared to non-Tg mice, indicating greater calpain activity in the presence of amyloid pathology (Fig. 2B and C). To determine if increased CFP–PLFAAR–YFP cleavage occurred in DNs, we quantified FRET between CFP and YFP in neuronal soma and DNs of fixed brain sections from the same mice. First, we validated effective FRET between CFP and YFP in fixed HEK293 cells and fixed 5XFAD brains by photobleaching the acceptor (YFP) and observing an increase in fluorescence of the donor (CFP) and a decrease of FRET (Supplemental Fig. 3B and C, respectively). Finally, we determined FRET:YFP ratio (Fig. 2D, E) in neuronal soma and DNs of 5XFAD mice expressing CFP–PLFAAR–YFP, demonstrating that FRET was decreased in DNs compared to the cell body. Images depicting a scaled ratio of FRET:YFP intensity visualized differences in calpain activity between neuron cell bodies and DNs (Fig. 2D). CFP images, which showed better resolution of individual DNs and cell bodies, were used to select regions of interest for quantification. Thiazine red staining marked plaque locations, so only DNs in the proximity of plaques were selected (Supplemental Fig. 3D). Together, these results show that calpain activity is elevated in DNs, which may contribute to pathologic fragmentation of tau.

### Membrane repair protein Annexin A6 localizes to the plasma membrane of neurons in normal brain

We hypothesized that DNs form due to membrane damage from contact with plaque-associated Aβ, which leads to increased calcium influx and calpain and kinase activation, as well as microtubule depolymerization, impaired axonal transport, and pathologic tau generation. If true, then enhanced membrane repair should reduce Aβ-associated DNs and pathologic tau. Annexin A6 functions to repair damage to plasma membranes in cells, especially those that undergo mechanical stress, such as muscle cells, or large cells with a high surface area, like neurons. We therefore investigated the possibility of enhancing membrane repair with annexin A6 to decrease the formation of dystrophic neurites.

**Fig. 3:**
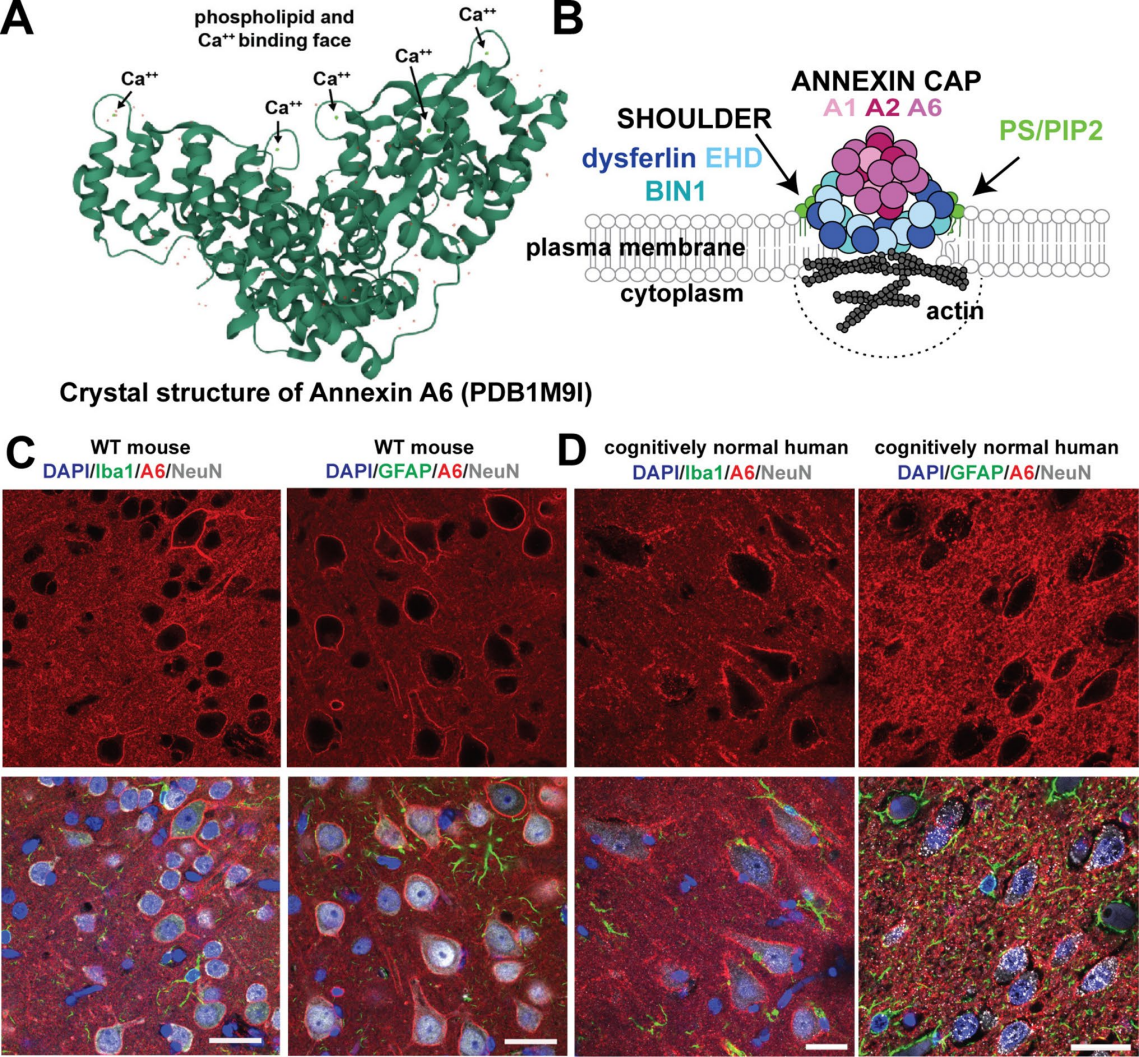
Annexin A6 localizes to neuronal plasma membrane in mouse and human brain. **A** Annexin A6 crystal structure. Arrows labeled “Ca^++^” point at green dots that represent bound calcium molecules. **B** Schematic of annexin A6 repair cap complex following membrane injury. PS, phosphatidylserine; PIP2, phosphatidylinositol biphosphate. **C, D** Wild-type (WT) mouse (**C**) and cognitively normal human (**D**) brain sections immunostained for annexin A6 (red), NeuN (gray), Iba1 (green), GFAP (green), and DAPI (blue)

First, we determined the cellular localization of endogenous annexin A6 in the brain using a specific antibody (rabbit monoclonal Abcam ab199422). We immunostained mouse and human brain sections and found annexin A6 localization in neurons, but not microglia or astrocytes, of wild-type mice (Fig. 3C) and cognitively normal aged humans (Fig. 3D). Most striking is the strong annexin A6 localization at the plasma membrane in both human and murine neurons.

Annexins A1 and A2 play secondary roles in membrane repair in muscle, adding to the repair cap after the recruitment of annexin A6 (Fig. 3B), and therefore may participate in membrane repair in neurons. To investigate this, we investigated single-cell RNA sequencing data from the Human Protein Atlas (https://www.proteinatlas.org/), which indicates high levels of annexin A6 in neurons. Conversely, annexins A1 and A2 are found in endothelial cells and pericytes in the brain, with very low levels in some excitatory neurons (A1) and inhibitory neurons (A2) (Supplemental Fig. 4A–C). Our immunostaining confirmed predominant localization of annexin A2 in vasculature and choroid plexus with no visible staining in neurons (Supplemental Fig. 4D, E), as previously reported [122]. Together, our results demonstrate that annexin A6 strongly localizes to neuronal membranes, and is a prime candidate for neuronal membrane repair in the brain.

**Fig. 4:**
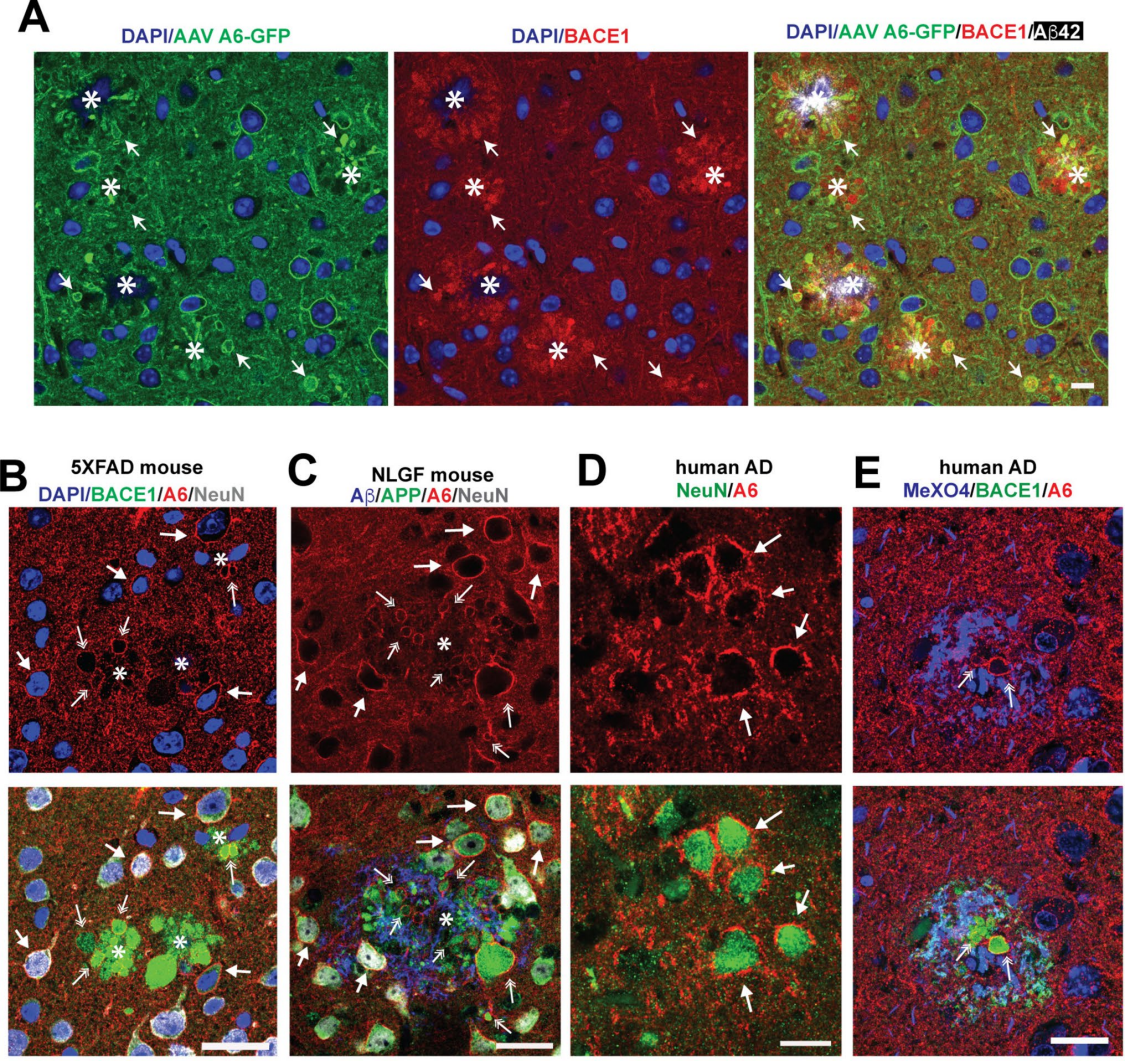
Overexpressed and endogenous A6 localizes to dystrophic neurite membranes. **A** Cortical section of 5XFAD mouse transduced with AAV expressing A6-GFP driven by the neuron-specific synapsin promoter and immunostained for A6-GFP (anti-GFP, green), BACE1 (red), Aβ42 (white), and DAPI (blue) shows A6-GFP localized to membranes of neuron soma and BACE1+ DNs (arrows); *plaques; Bar=10μm. **B**, **C** 5XFAD (**B**) and APP-NLGF (**C**) cortical mouse brain sections immunostained for annexin A6 (red), BACE1 (green), APP (green), Aβ (blue), NeuN (gray), and DAPI (blue). We used the APP-NLGF knock-in mouse model of amyloid pathology to validate results obtained with 5XFAD mice. **D**, **E** Human AD hippocampal brain sections immunostained for annexin A6 (red), BACE1 (green), MeX04 (amyloid stain; blue), and NeuN (green). *plaque cores; double arrows, DNs; arrows, neurons. Bars=10μm (A), 25μm B-E)

### In the presence of amyloid plaques, endogenous and overexpressed annexin A6 localizes to dystrophic neurites

The strong prevalence of annexin A6 over A1 and A2 in neurons, along with the observation that A6 localizes to sites of injury in neurons [29] and is the key factor for initiation of membrane repair in myofibers [30, 31], led us to focus on annexin A6 expression as a potential approach for reducing Aβ-associated membrane damage, DN formation, and pathologic tau generation in AD. To test whether annexin A6 could reduce DNs, we used a somatic brain transgenesis approach [71] to express syn-GFP AAV or syn-A6-GFP in the neurons of mice throughout their lifetime. On P0, we performed intracerebroventricular injections of pups with either syn-GFP AAV or syn-A6-GFP AAV8, then aged them to 4.5 months and collected brain tissue. Immunoblot analysis showed that A6-GFP was expressed ~8 fold higher than endogenous A6 (Suppl. Fig. 5 A, B), which mice tolerated well with no obvious ill effects, and protein expression was widespread through the cortex and hippocampus (Suppl. Fig. 5C). GFP fluorescence revealed A6-GFP expression in neurons and DNs (Fig. 4A). A6-GFP colocalized with BACE1 in DNs, indicating A6-GFP reached the correct location to repair potential membrane damage in DNs. AAV-mediated A6-GFP overexpression recapitulated the localization of endogenous A6, which was also found in membranes of neurons and DN (marked by BACE1 or APP) in 5XFAD transgenic (Fig. 4B) and APP-NLGF knock-in (Fig. 4C) amyloid model mice, and brains from AD patients (Fig. 4D, E) where it could play a role in amyloid-induced membrane repair.

### AAV-mediated expression of annexin A6 reduces dystrophic neurites

**Fig. 5:**
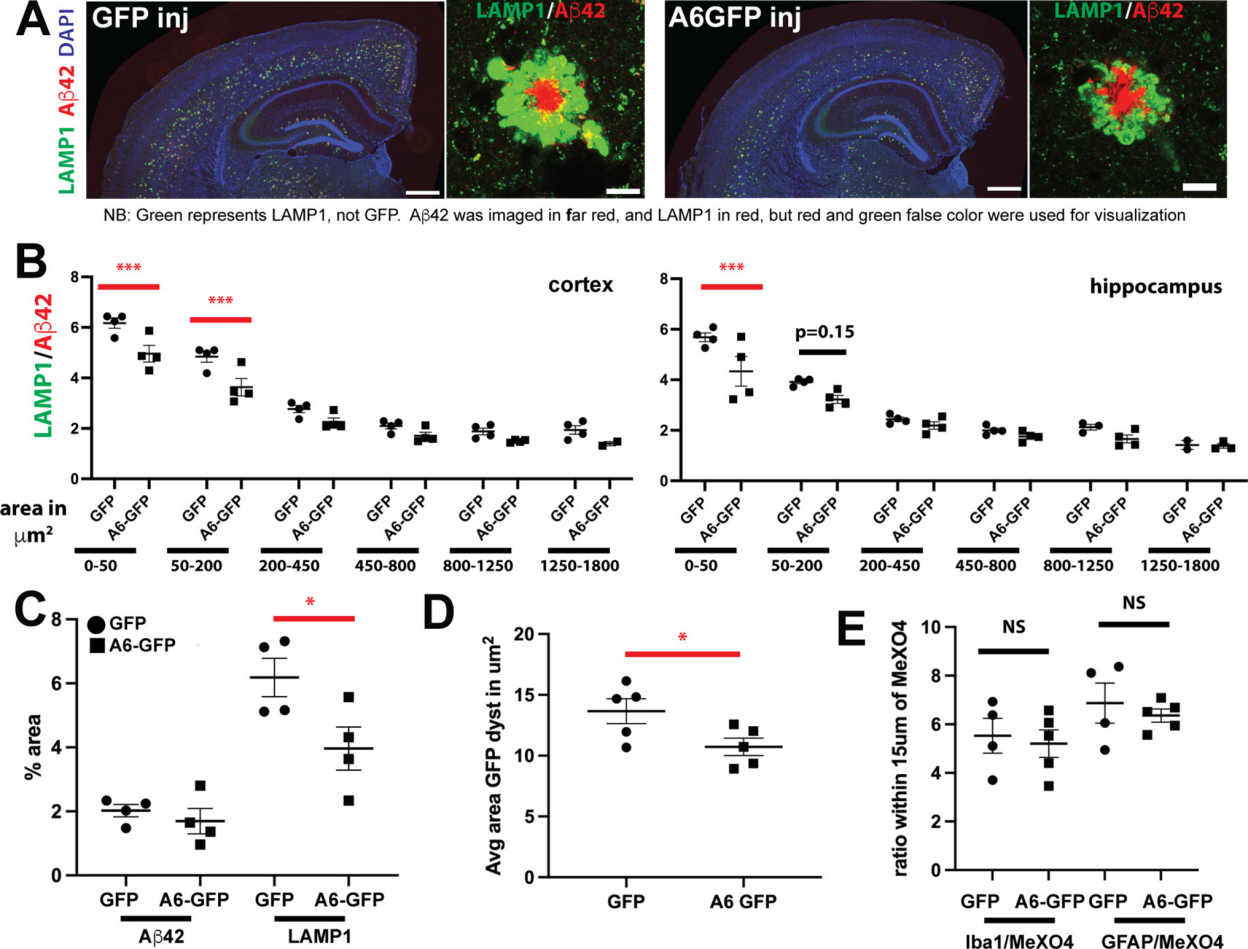
Annexin A6-GFP reduces dystrophic neurites without affecting Aβ deposits or glia in 5XFAD brain. **A** Coronal brain sections of 5XFAD mice transduced with AAV expressing GFP alone or A6-GFP driven by the neuron-specific synapsin promoter and immunostained for Aβ42 (red), LAMP1 (green), and DAPI (blue). Note the smaller LAMP1+ dystrophic neurite halo surrounding the amyloid plaque in A6-GFP expressing 5XFAD mice (far right). Bars=500 μm, left panels; bars=10 μm, right panels. **B** LAMP1/Aβ42 ratio of amyloid plaques in cortex (left) and hippocampus (right) of 5XFAD mice expressing GFP alone (*n* = 4) or A6-GFP (*n* = 4) binned by plaque core area with the indicated ranges in μm^2^. Expression of A6-GFP significantly reduced LAMP1/Aβ42 ratio in smaller, faster growing plaques in the size ranges of 0–50 and 50–200 μm^2^. ***, *p* < 0.001. **C** %Aβ42+ (left graph plots) and %LAMP1+ (right graph plots) areas are unchanged and significantly reduced, respectively, in 5XFAD mice expressing A6-GFP compared to GFP alone. *p < 0.05. **D** Endogenous GFP fluorescence was imaged using confocal microscopy, and ImageJ used to quantify size of GFP+ DNs. Average area of GFP+ DNs is significantly reduced in 5XFAD mice expressing A6-GFP compared to GFP alone. *, p<0.05. **E** Ratios of Iba1 (left graph plots) and GFAP (right graph plots) to amyloid as measured by MeX04 staining within 15 μm of plaque cores are unchanged in 5XFAD mice expressing A6-GFP compared to GFP alone, indicating A6-GFP causes no change in microglia or astrocytes near plaques. In all graphs, each point represents an individual animal

To determine the effects of annexin A6-GFP on amyloid pathology and DN formation, brain sections from A6-GFP or GFP-alone transduced mice were stained with antibodies against Aβ42 and LAMP1, the latter recognizing lysosomal and late endosomal compartments as well as autophagic organelles that accumulate to high levels in DNs, making it a commonly used DN marker [20, 21, 46] (Fig. 5A). We used Nikon NIS-Elements software to define each plaque and its associated DN halo and determine the ratio of the respective areas stained for LAMP1 and Aβ42 (Fig. 5B). Smaller amyloid plaques grow at faster rates compared to larger plaques and have proportionally greater amounts of neuritic dystrophy [20], so we stratified plaques according to size, based on staining with an anti-Aβ42 antibody, before averaging LAMP1:Aβ42 ratios. As expected, smaller plaques had a higher LAMP1:Aβ42 ratios than larger plaques (Fig. 5B). Importantly, we observed a significant decrease of the LAMP1:Aβ42 ratio of smaller plaques in the hippocampus and cortex of 5XFAD brains injected with A6-GFP AAV compared to those injected with GFP AAV (Fig. 5B). Smaller plaques exhibit the most rapid growth and cause the greatest neurotoxicity [20], so reducing DNs around smaller plaques is likely to have beneficial effects. The distribution of plaque sizes was equivalent in A6-GFP and GFP expressing animals (Suppl. Fig. 5D). The total percent area positive for LAMP1 in the cortex was significantly decreased in A6-GFP AAV-injected 5XFAD brains, while that of Aβ42 was unchanged (Fig. 5C), indicating that A6-GFP specifically reduced DNs with minimal effects on Aβ deposits.

Using higher magnification confocal microscopy images, we observed that the area of individual GFP-positive DNs was significantly reduced in A6-GFP AAV-injected mice compared to those injected with GFP AAV (Fig. 5D). A6-GFP did not affect microglia or astrocytes around plaques, as the ratio of microglial marker Iba1 and astrocyte marker GFAP to the amyloid stain MeX04 in the area within 15μm of the plaque core was unchanged (Fig. 5E). Together, these results strongly suggest that annexin A6 overexpression reduces the formation of DNs, likely via its membrane repair function, but does not affect Aβ pathology.

### Tau phosphorylated at Thr181 accumulates in dystrophic neurites and is reduced by annexin A6 overexpression

**Fig. 6:**
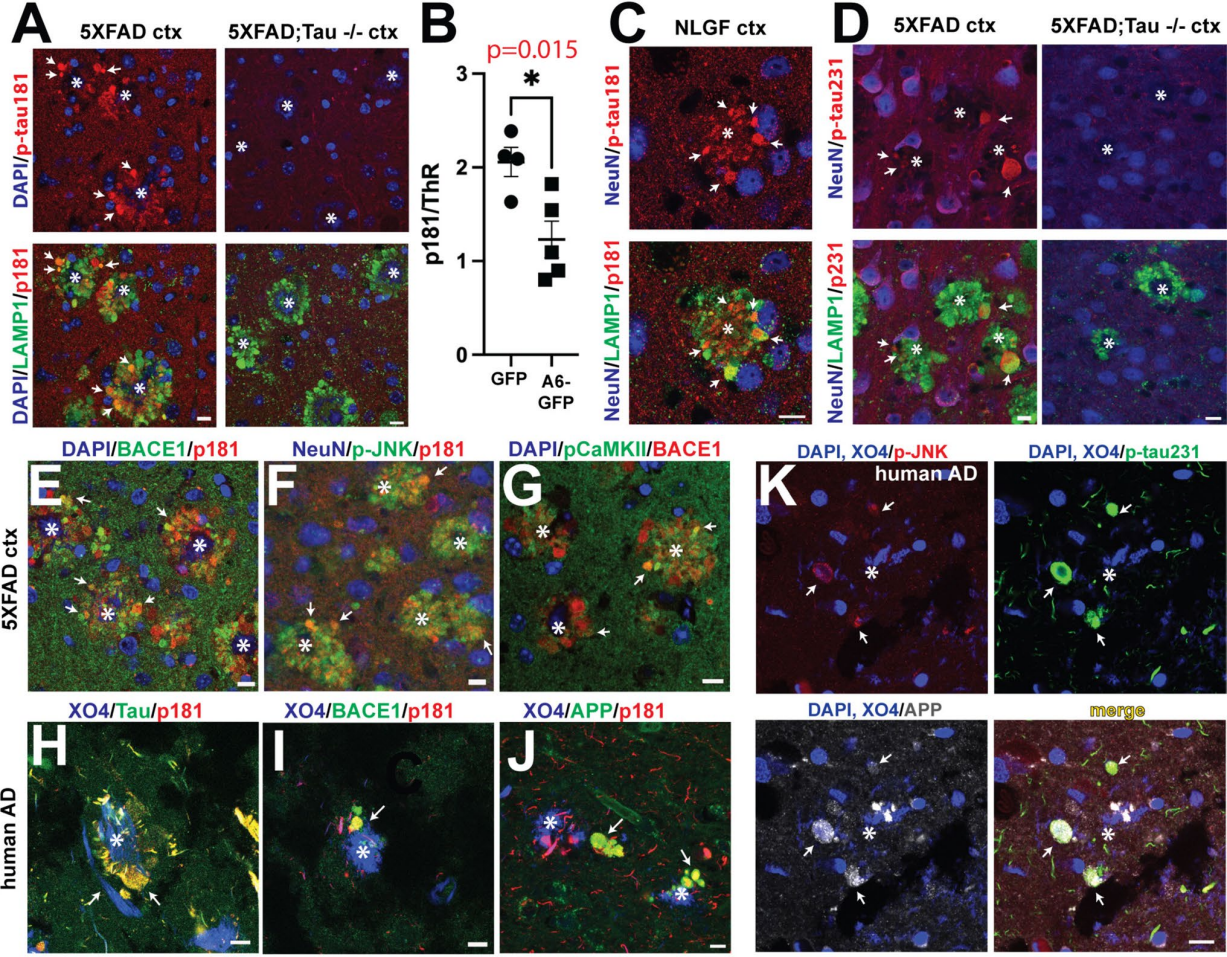
p-tau181, p-tau231, and phosphorylated tau kinases increase in 5XFAD, APP-NLGF, and human AD dystrophic neurites, while annexin A6-GFP expression decreases p-tau181. **A** 5XFAD (left) and 5XFAD;Tau^-/-^ (right) cortex (ctx) sections immunostained for p-tau181 (red), LAMP1 (green), and DAPI (blue). In 5XFAD brain, p-tau181 accumulates in LAMP1+ DNs in contact with amyloid plaques. Note that 5XFAD;Tau^-/-^ brain forms amyloid plaques and LAMP1+ DNs, but that 5XFAD;Tau^-/-^ DNs lack p-tau181 accumulation. **B** p-tau181:Thiazine red (ThR; amyloid stain) ratio in 5XFAD mice transduced with AAV expressing GFP or A6-GFP driven by the neuron-specific synapsin promoter. Note that p-tau181:ThR ratio is significantly decreased in 5XFAD mice expressing A6-GFP. **C** APP-NLGF cortex section immunostained for p-tau181 (red), LAMP1 (green), and NeuN (blue). The accumulation of p-tau181 in DNs of APP-NLGF appears very similar to that of 5XFAD mice. **D** 5XFAD (left) and 5XFAD;Tau^-/-^ (right) cortex sections immunostained for p-tau231 (red), LAMP1 (green), and NeuN (blue). **E**–**G** 5XFAD cortex sections immunostained for p-tau181 (red), p-JNK (green), p-CaMKII (green), BACE1 (red), NeuN (blue), and DAPI (blue). **H**–**J** AD hippocampal sections immunostained for p-tau181 (red), total tau (Tau5, green), MeX04 (amyloid stain, blue), BACE1 (green), and APP (green). **K** AD hippocampal section immunostained for *p*-tau231 (green), p-JNK (red), APP (gray), MeX04 (blue), and DAPI (blue). *, plaque cores; arrows, DNs. Bars=10 μm. Images representative from *n* = 3 mice

Previous work has suggested that plaques and associated neuritic dystrophy play a key role in pathologic AD tau seeding and spreading [53, 72, 89]. When tau seeds purified from human AD brain are injected into amyloid mouse model brains, tau spreading, as detected by antibody AT8, positively correlates with the amount of plaques and the degree of neuritic dystrophy around plaques, with AT8 positivity appearing specifically in DNs rather than cell bodies [53, 72, 89]. CSF and plasma biomarkers p-tau181 and p-tau231 correlate significantly with Aβ pathology and predict AD onset years in advance [6, 7, 79, 107]. We therefore hypothesized that these early markers of Aβ pathology would be present in DNs. Indeed, using specific antibodies for p-tau181 (Fig. 6A, C) and p-tau231 (Fig. 6D), we observed that p-tau181 and p-tau231 accumulated in DNs of 5XFAD and APP-NLGF brains, as shown by colocalization with LAMP1 and/or BACE1. No signal for p-tau181 or p-tau231 was detected in 5XFAD;Tau^-/-^ brain (Fig. 6A, D). As we show here, and others have reported [92, 96], amyloid mouse models still have robust DN formation in the absence of tau, clearly indicating that tau is not required for generating DNs. Interestingly, p-tau231 and p-tau181 had different localization patterns. P-tau181 appeared in mossy fibers and DNs in both wild-type and 5XFAD mice (Suppl. Fig. 6A, B), while p-tau231 localization was more somato-dendritic (Suppl. Fig. 6C, D). These observations suggest that p-tau181 is predominantly found in axonal compartments compared to p-tau231. However, p-tau231 does appear in DNs, but less frequently than p-tau181. Moreover, these results strongly support the hypothesis that amyloid deposition leads to aberrant tau phosphorylation via the formation of DNs.

Since annexin A6-GFP overexpression reduced DNs in 5XFAD brain (Fig. 5), we hypothesized that A6-GFP would also decrease phosphorylated tau associated with DNs. Indeed, we observed a significant reduction in the ratio of p-tau181 to amyloid in 5XFAD mice injected with syn-A6-GFP AAV compared to those injected with syn-GFP AAV (Fig. 6B). We hypothesized that increased kinase activity in DNs could be responsible for p-tau accumulation. Activated, phosphorylated forms of two kinases, CaMKII and JNK, known to phosphorylate p-tau181, p-tau217, and p-tau231 [78, 117], colocalized with p-tau181 in 5XFAD DNs (Fig. 6E–G). In human AD brain, p-tau181 colocalized with total tau, BACE1, and APP in DNs around plaques (Fig. 6H–J) and p-tau 231 colocalized with p-JNK and the DN marker APP (Fig. 6K). Co-occurrence of p-tau with p-JNK and p-CaMKII in DNs does not demonstrate a physical interaction in these structures, but does suggest that DNs could be sites of early tau phosphorylation, especially p-tau181, which is used as an early CSF and plasma biomarker that indicates amyloid pathology and predicts AD [19, 65, 107].

### Reduced annexin A6 function results in more dystrophic neurites and increased p-tau181

**Fig. 7:**
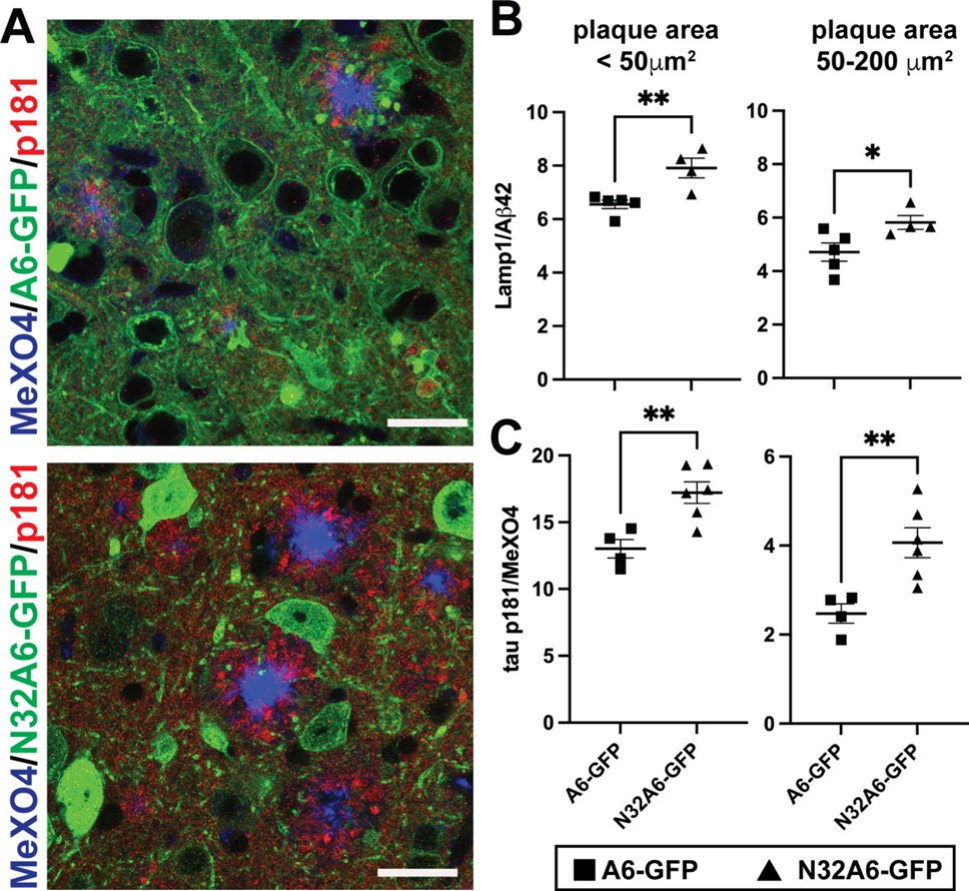
Dominant negative annexin N32 A6-GFP increases dystrophic neurites and p-tau181 in 5XFAD brain. **A** Cortical sections of 5XFAD mice transduced with AAV expressing annexin A6-GFP or dominant-negative A6 truncation, N32 A6-GFP driven by the neuron-specific synapsin promoter immunostained for GFP (green), p-tau181 (red), and MeX04 (amyloid stain, blue). Note the mislocalization of N32 A6-GFP away from the plasma membrane toward the cytoplasm and nucleus in N32 A6-GFP compared to A6-GFP expressing neurons. Bar=25 μm. **B** LAMP1:Aβ42 ratio of amyloid plaques in the cortices of A6-GFP and N32 A6-GFP expressing 5XFAD mice binned by plaque core area with the indicated ranges in μm^2^. Smaller, faster growing plaques in the size ranges < 50 μm^2^ (left) and 50–200 μm^2^ (right) of N32 A6-GFP expressing mice had significantly increased LAMP1:Aβ42 ratio compared to that of A6-GFP expressing mice. **C** The p-tau181:MeX04 ratio of amyloid plaques in the cortices of A6-GFP and N32 A6-GFP expressing 5XFAD mice binned by plaque core area with the indicated ranges in μm^2^ (<50 μm^2^, left; 50–200 μm^2^, right). As with LAMP1/Aβ42 ratio (**B**), p-tau181:MeX04 ratio was significantly increased in smaller, faster growing plaques of N32 A6-GFP compared to A6-GFP expressing mice. **p < 0.01, *p < 0.05. *n* = 4–6 mice

To determine the effects of reduced annexin A6 function on DNs, we utilized a naturally occurring annexin A6 truncation (p.N32*) generated by altered splicing that introduces a premature stop codon, which results in a protein lacking the last four of the eight annexin repeats [108]. Even at low levels, A6 N32 protein exerts strong dominant-negative effects, blocking the assembly of the repair cap by preventing the recruitment of full-length annexin A6 and other repair cap components to the site of membrane damage [31, 108]. Using the P0 intracerebroventricular injections of AAV8 described previously, we overexpressed an N32 A6-GFP fusion protein in neurons of 5XFAD mice and found a loss of membrane localization compared to A6-GFP (Fig. 7A), which was also observed in non-transgenic mice (Suppl. Fig. 7). The average LAMP1:Aβ42 ratio was significantly increased in mice overexpressing N32 A6-GFP compared to those expressing A6-GFP (Fig. 7B). Additionally, the ratio of p-tau181 to amyloid as measured by MeX04 staining was significantly elevated by N32 A6-GFP (Fig. 7C). Together, the increase and decrease of DNs and p-tau181 caused by expression of N32 A6-GFP and A6-GFP, respectively, strongly suggest an important role for annexin A6 in the repair of Aβ-induced membrane damage and DN formation.

### Extracellular recombinant annexin A6-GFP localizes to membranes of dystrophic neurites

**Fig. 8:**
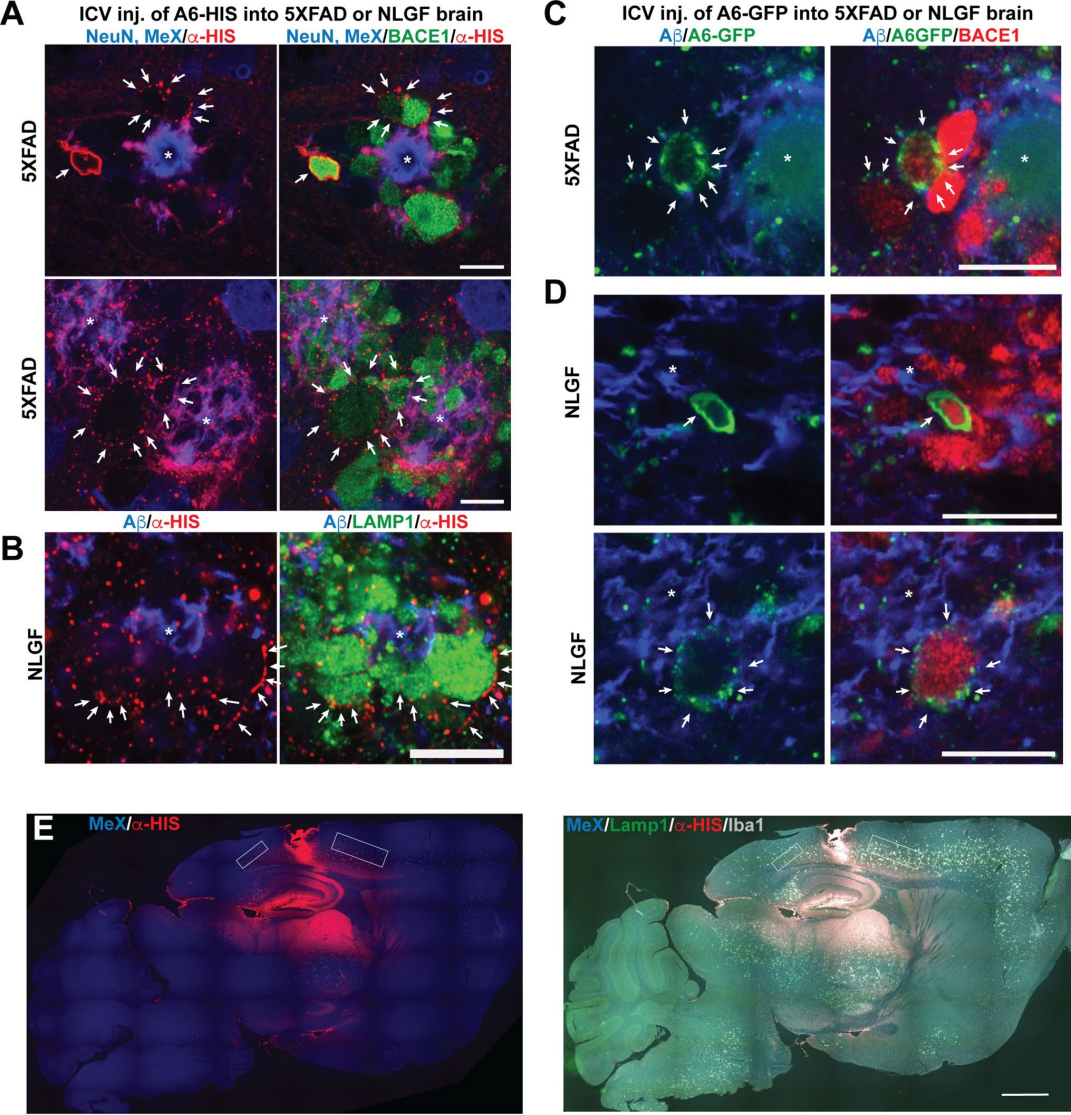
Recombinant annexin A6 localizes to dystrophic neurites following intracerebral ventricular injection in 5XFAD and APP-NLGF mice. **A-D** Cortical sections from 5XFAD (**A**) and APP-NLGF (**B**) mice that received a single intracerebral ventricular (ICV) injection of recombinant annexin A6-HIS (3.3 mg/kg) or A6-GFP (0.9 mg/kg; **C** 5XFAD; **D** NLGF) and brains harvested 3 h later. Sections were immunostained for α-HIS (red), NeuN (blue), MeX04 (amyloid stain, blue), BACE1 (red), LAMP1 (green), and Aβ (3D6, blue). GFP fluorescence of A6-GFP was imaged in (**C**) and (**D**). Both recombinant A6-HIS and A6-GFP localized to membrane puncta and whole membranes on BACE1+ and LAMP1+ dystrophic neurites (arrows) in 5XFAD and APP-NLGF mice, indicating the presence of membrane damage. *, plaque cores; Bars=10μm. Representative from *n* = 2–5 mice per genotype, and protein injection. **E** Low magnification images of brain section from 5XFAD mouse after intracerebral ventricular (ICV) injection of recombinant annexin A6-HIS (3.3 mg/kg), immunostained with MethoxyX04 (blue) for amyloid, α-HIS (red), LAMP1 (green), and Iba1 (white) to demonstrate the spread of A6-HIS after three hours. White boxes indicate approximate areas at edge of injection spread selected for 60x confocal imaging in similar sections for A through D. Scale bar = 1000 μm

Previous work in skeletal muscle demonstrates that both intracellular annexin A6 overexpression and extracellular rA6 can enhance membrane repair [30]. Intracellular and extracellular annexin A6 promote myofiber membrane resealing after laser injury and reduce dye influx to equivalent amounts. Additionally, rA6 is also effective in acute (lytic damage from cardiotoxin injection) and chronic (muscular dystrophy due to sarcoglycan mutation) muscle injury models [30]. These results strongly suggest that rA6 could be an effective therapeutic agent, since it can home to sites of membrane damage from the extracellular space allowing exogenous administration of rA6. We previously published that exogenous rA6 localized to sites of laser damage on primary neurons [29], but we wanted to test whether extracellular rA6 would home to DNs *in vivo*. To do so, we administered a single stereotaxic injection of recombinant A6-HIS (Fig. 8A, B) or A6-GFP (Fig. 8C, D) into the lateral ventricle of 5XFAD (Fig. 8A,C, E) or APP-NLGF (Fig. 8B, D) mice and harvested brains 3 hours later. In the immediate vicinity of the needle track (Fig. 8E), A6-HIS strongly bound to damaged membranes resulting from the injection. Further from the injection site away from needle injury (boxed regions in Fig. 8E), immunofluorescence confocal microscopy revealed that both extracellular A6-HIS and A6-GFP localized to plasma membranes of BACE1- and LAMP1-positive DNs (Fig. 8A, B). In some cases (top panel Fig. 8A), rA6 completely coated the DN membrane, suggesting extensive externalization of phosphatidylserine, which could target the DN for engulfment and clearance by microglia or indicate cell death [101]. However, in most cases, we observed rA6 puncta more indicative of localized regions of membrane disruption. Although single stereotaxic injections of rA6 are unlikely to have long-term therapeutic effects, they demonstrate that extracellular rA6 can in principle target damaged membranes on dystrophic neurites near amyloid plaques as a proof of concept for an AD therapy. Together, our results support further investigation of extracellular administration of rA6 as a potential novel therapeutic approach to limit Aβ-induced membrane damage for the prevention of dystrophic neurites in AD, which could have beneficial effects on tau pathology.

## Discussion

In this work, we have demonstrated a role of annexin A6 in reducing DNs in the 5XFAD amyloid mouse model. We show that endogenous annexin A6 is found at the plasma membrane in neurons of mice and humans. In AD patients or AD mouse models, A6 localizes to the membrane of DNs in contact with amyloid plaques. Overexpression of wild-type annexin A6-GFP decreased DNs and p-tau181, while expression of dominant-negative truncated N32 A6-GFP increased DNs and p-tau181. We also demonstrate elevated calcium levels and calpain activity in DNs of 5XFAD mice. Additionally, we observed the presence of phosphorylated, active forms of tau kinases, such as p-CaMKII and p-JNK in DNs, which in combination with active calpain could lead to increased phosphorylation and cleavage of tau that promote aggregation-prone tau and tau seeding and spreading. Finally, we demonstrate that, as in other systems (muscle, heart, and primary neurons), exogenous recombinant annexin A6 can localize to and bind damaged neuronal membranes from the extracellular space, opening the possibility for a protein-based AD therapy.

The calcium hypothesis of AD posits that dysregulation of calcium homeostasis is the point of convergence of the many risk factors and molecular mechanisms that lead to development of AD and its associated neurodegeneration [3, 67]. Our work in this paper supports the calcium hypothesis, showing definitively that baseline resting calcium is elevated in DNs contacting Aβ plaques. Recent work demonstrates that DNs are detrimental, preventing effective transmission of action potentials and leading to loss of synaptic function [61, 120], which can contribute to cognitive decline. We further support the detrimental role of calcium influx in DNs by showing downstream changes, such as increased calpain activity, accumulation of phospho-tau epitopes, and phosphorylated forms of known tau kinases JNK and CaMKIIα, which is phosphorylated in the presence of high calcium [100].

As the “first responder” to calcium influx following membrane damage, annexin A6 plays the key role to initiate the formation of the membrane repair complex [30, 31]. Annexin A6 expression levels do not appear to be altered in the brains of either 5XFAD mice or AD patients (data not shown), but examples exist of recombinant and overexpressed annexins being used to protect against injury or damage in the brain. Previously, it was shown that recombinant annexin A6 or A5 reduced dye leakage from liposomes induced by Aβ and various membrane disruptors [23]. Injection of exogenous recombinant annexin A2 was protective in a traumatic brain injury model by decreasing blood–brain barrier (BBB) permeability and promoting angiogenesis [17], and annexin A5 protected choroid plexus cells from damage by Aβ42 [8]. Injection of human recombinant annexin A1 acutely decreased BBB permeability in young 5XFAD and Tau P301L mice, as well [95], further suggesting beneficial effects of annexins in neurological disease. Lentiviral overexpression of annexin A6 in mice improved outcomes after transient focal ischemia, minimizing loss of synaptic proteins [113]. Although annexin A6 constitutive null mice were shown to have normal immune and cardiac function [52], more recently, they were found to have increased sensitivity to painful mechanical stimuli due to loss of annexin A6 interaction with the Piezo2 channel [94]. These data support the idea that annexins A1, A2, A5, and A6 all play a role in protecting the brain.

In muscle, it is clear that annexins A1 and A2 work with annexin A6 in membrane repair, but in most neurons, little or no annexins A1 and A2 are expressed to interact with annexin A6 to form the membrane repair cap. Annexins A1 and A2 are expressed in the brain, but primarily in endothelial cells (Supplemental Fig. 4). It is known that annexins, such as A1 and A2, are found extracellularly, likely secreted through non-canonical pathways [93], and recent publications report that annexins A2 and A6 are found at high levels in the extracellular matrix of muscle where they promote myoblast motility [76]. Annexins A7 and A11 are highly expressed in the majority of neuronal cell types, like annexin A6, where they localize to plasma and nuclear membranes (A11) or to cytoplasm (A7), so it is possible they could interact with annexin A6 to facilitate membrane repair. Annexins A11 and A7 are unique in the annexin family in having a long unstructured N-terminus [44], which for annexin A11 is involved in RNA binding. Annexin A11 tethers mRNA to lysosomes for transport in neurons, and mutations disrupting this function are associated with amyotrophic lateral sclerosis in humans [75, 85]. Annexin A7 recruits the Endosomal Sorting Complex Required for Transport III (ESCRT III) complex to sites of membrane damage and facilitates repair through calcium-triggered protein interactions [105], so annexin A7 could complement the repair functions of annexin A6.

While membrane repair is a key function of annexin A6, it has been observed to play a role in other cellular processes, suggesting the possibility that annexin A6 may have pleiotropic effects in neurons. It has been reported that the extreme N-terminus of tau can interact with annexins A2 and A6 core domains in calcium-dependent manner, and that the annexin A6–tau interaction affects tau microtubule binding and sorting into the axon [41, 42]. This latter notion is supported by the localization of annexin A6 to the axon initial segment (AIS), where it seems to promote axon branching [116]. Additionally, a recent study profiling the tau interactome in typical compared to rapidly progressing AD found increased levels of annexin A6 in the tau interactome of typical AD, suggesting that the annexin A6–tau interaction may protect against tau pathology, or that higher levels of annexin A6 improve membrane repair leading to slower AD progression [118]. Finally, two recent large genome-wide association studies (GWAS) have reported protective associations with AD in *TNIP1* within 50 kb of *ANXA6* [9, 114]*.* While the lead SNP was located in a different gene, the partial contribution of *ANXA6* to this signal cannot be completely ruled out.

In our work, it is possible that annexin A6–tau interactions may have played a role in decreasing tau phosphorylation in DNs. Alternatively, overexpression of annexin A6 during postnatal developmental may have affected axon outgrowth, although we did not observe any differences in the brains of mice expressing either A6-GFP or GFP alone. In hepatocytes, annexin A6 is associated with membranes of endosomes, multivesicular bodies, and autophagosomes, where it likely plays a role in remodeling membrane architecture and promoting vesicle fusions [35, 36]. Therefore, although our results strongly suggest the mechanism of annexin A6 in reducing DNs and tau phosphorylation involves repair of Aβ-induced membrane damage, it is possible other annexin A6 mechanisms may be at play.

Our study has several limitations. Since recombinant annexin A6 (rA6) added exogenously to cells or tissue can home to sites of membrane damage and mediate repair from outside the cell, rA6 may have potential as a novel biologic therapy for AD. However, several issues need to be resolved before exogenous rA6 administration can be considered a viable therapeutic strategy. First, while our experiments demonstrate that genetically overexpressed annexin A6 from birth can reduce DNs and phosphorylated tau, it still must be shown that overexpression of annexin A6 at different stages of Aβ pathology can reduce the formation of DNs and phosphorylated tau. Additionally, although injected rA6 showed localization to the DN membranes, it is necessary to perform chronic administration to determine whether rA6 can protect against DNs and tau phosphorylation. Current work is focused on the effects of AAV-mediated annexin A6 overexpression in adult mice that have Aβ pathology, chronic rA6 delivery through osmotic mini-pumps, and development of methods for delivery of rA6 across the blood–brain barrier [115] to allow chronic peripheral administration of rA6 as a more clinically practical method of testing therapeutic applicability. Future work will also focus on testing the ability of overexpressed and exogenous annexin A6 to decrease DNs and prevent in amyloid mouse models the seeding and spreading of pathologic tau isolated from human AD brain. Further understanding of the role of Aβ-induced membrane damage in DN formation and how to prevent it promises to shed light on the pathologic linkage between Aβ and tau, one of the most profound mysteries of AD.

## Supplementary Information

Below is the link to the electronic supplementary material.

Supplementary file1 (TIF 19273 kb)

Supplementary file2 (TIF 24065 kb)

Supplementary file3 (TIF 10899 kb)

Supplementary file4 (TIF 14003 kb)

Supplementary file5 (TIF 4400 kb)

Supplementary file6 (TIF 12372 kb)

Supplementary file7 (TIF 15094 kb)

Supplementary file8 (DOCX 19 kb)
