# Supplementary figures and images for: Annexin A6 membrane repair protein protects against amyloid-induced dystrophic neurites and tau phosphorylation in Alzheimer’s disease model mice
Source: Acta Neuropathol. 2025 May 24;149(1):51. doi: 10.1007/s00401-025-02888-1 (PMC12103342; doi:10.1007/s00401-025-02888-1)

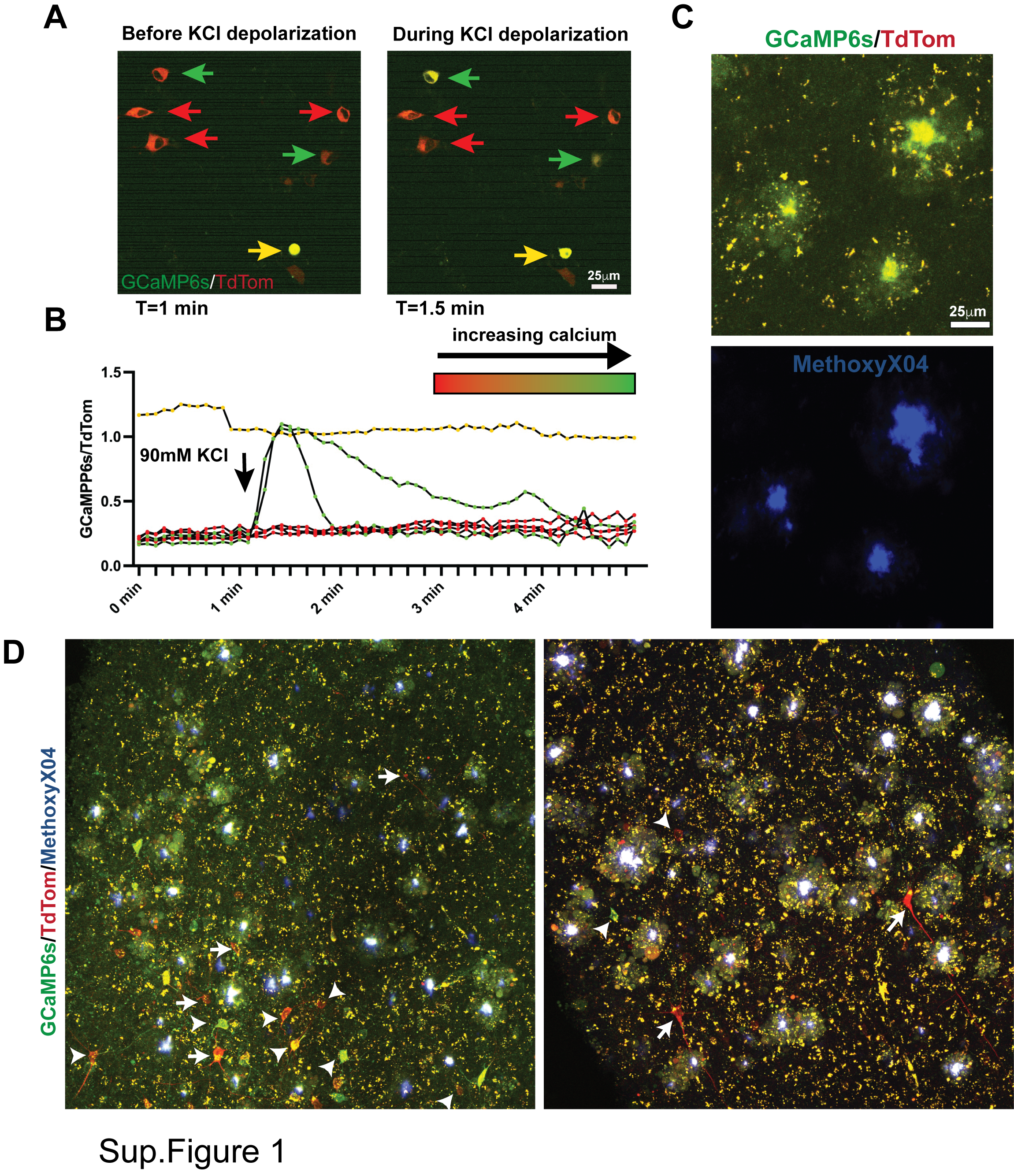

Supplement: Supplementary file 1 — Supplementary file1 (TIF 19273 kb) [file 401_2025_2888_MOESM1_ESM.tif]

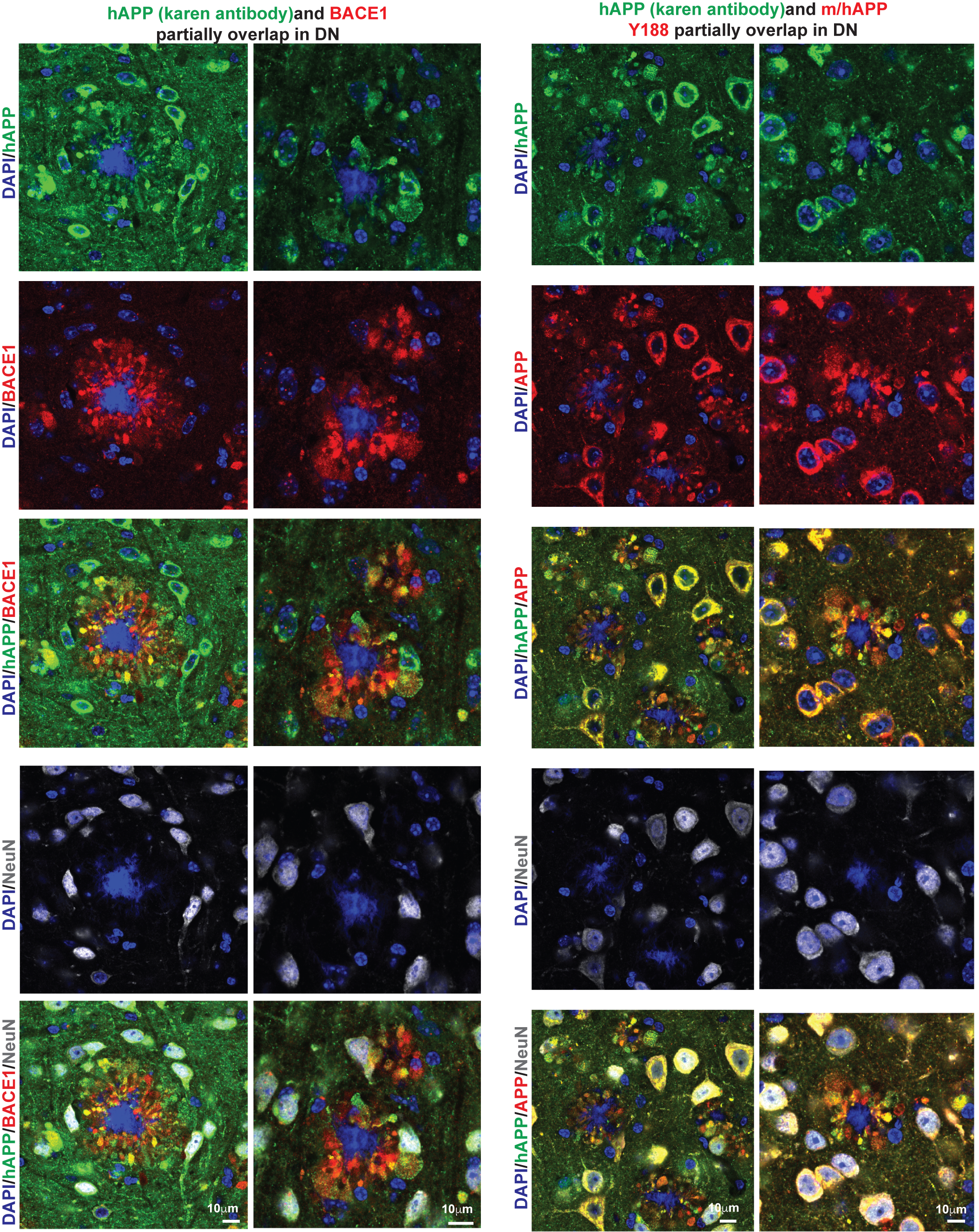

Supplement: Supplementary file 2 — Supplementary file2 (TIF 24065 kb) [file 401_2025_2888_MOESM2_ESM.tif]

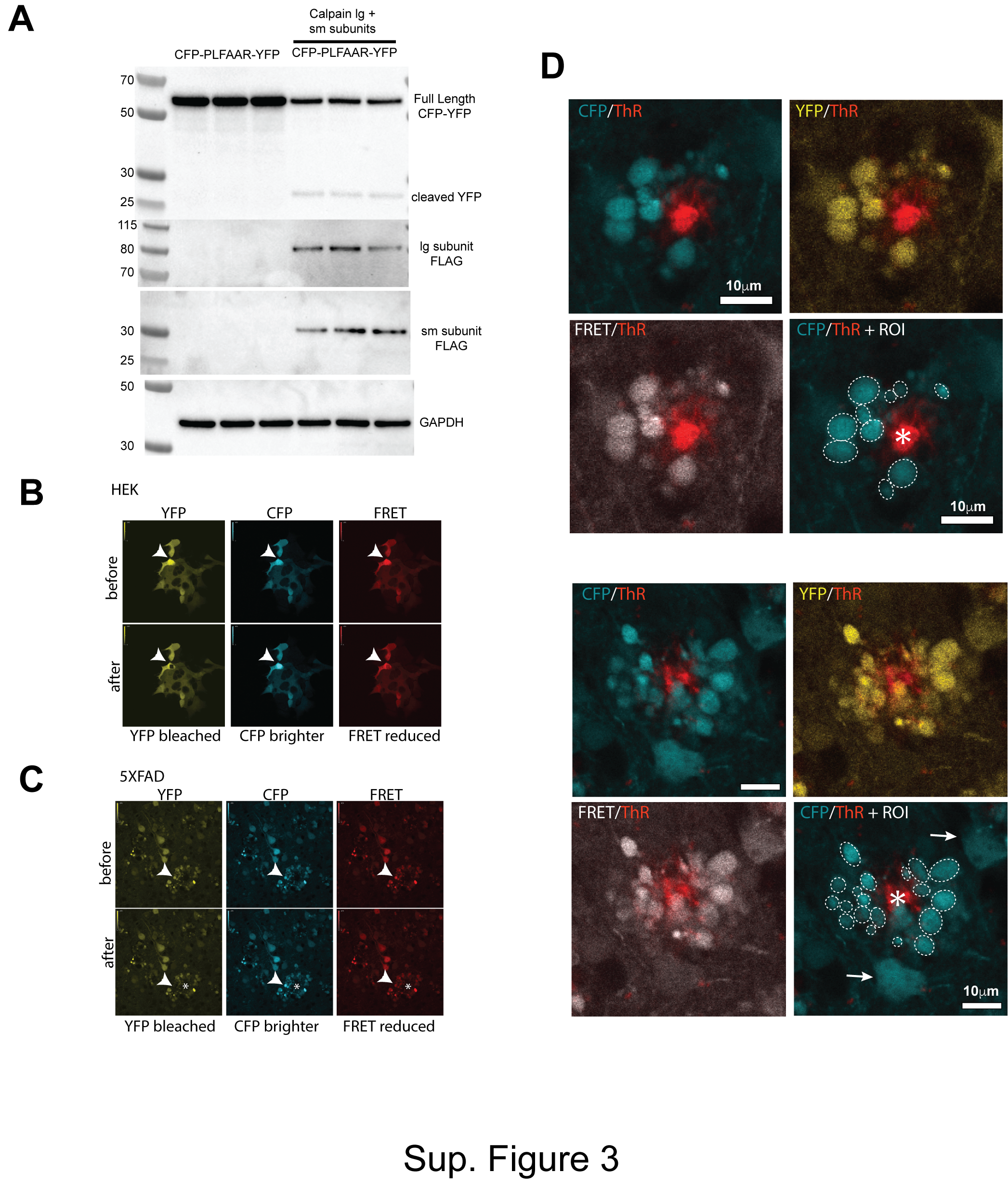

Supplement: Supplementary file 3 — Supplementary file3 (TIF 10899 kb) [file 401_2025_2888_MOESM3_ESM.tif]

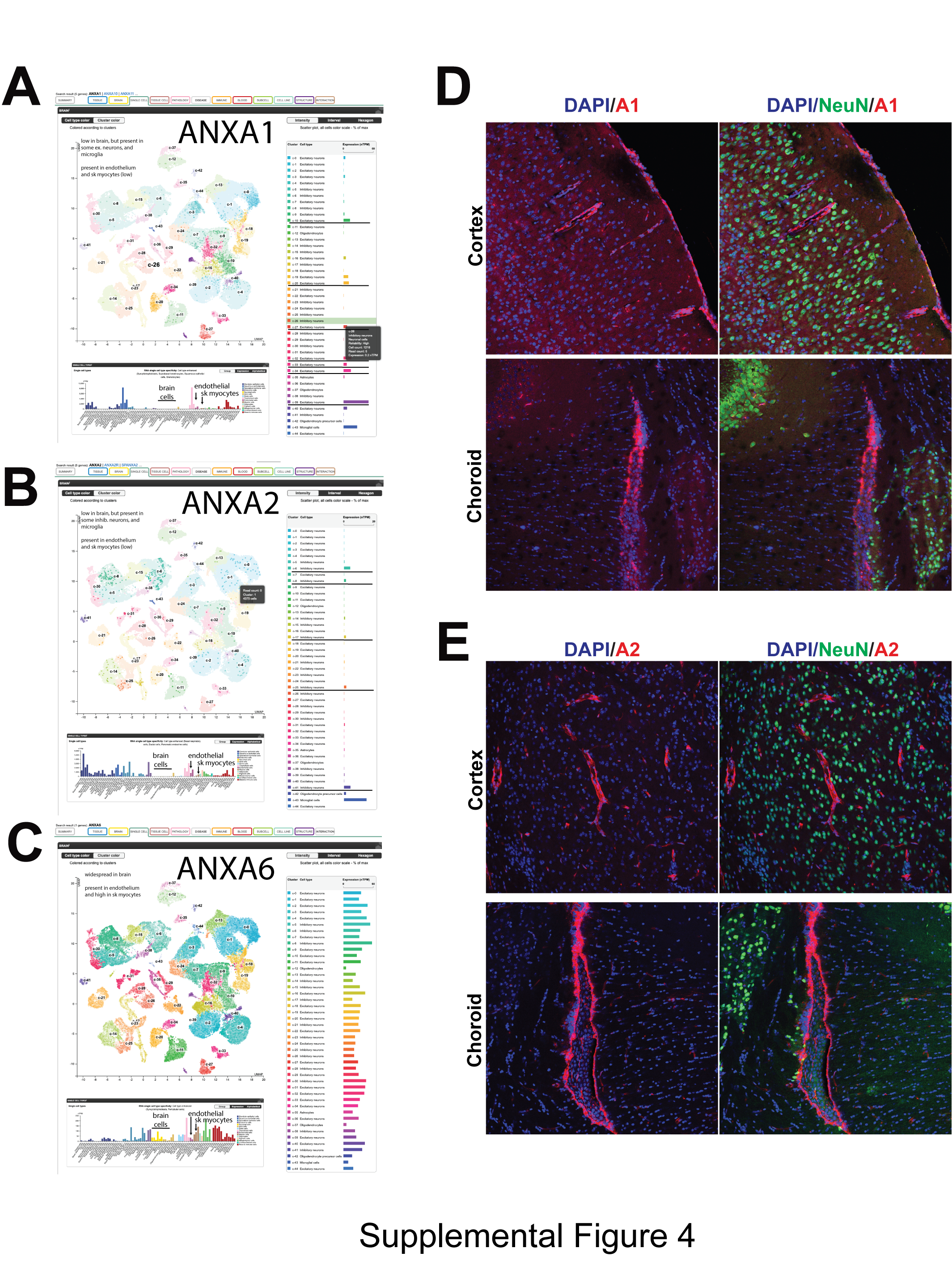

Supplement: Supplementary file 4 — Supplementary file4 (TIF 14003 kb) [file 401_2025_2888_MOESM4_ESM.tif]

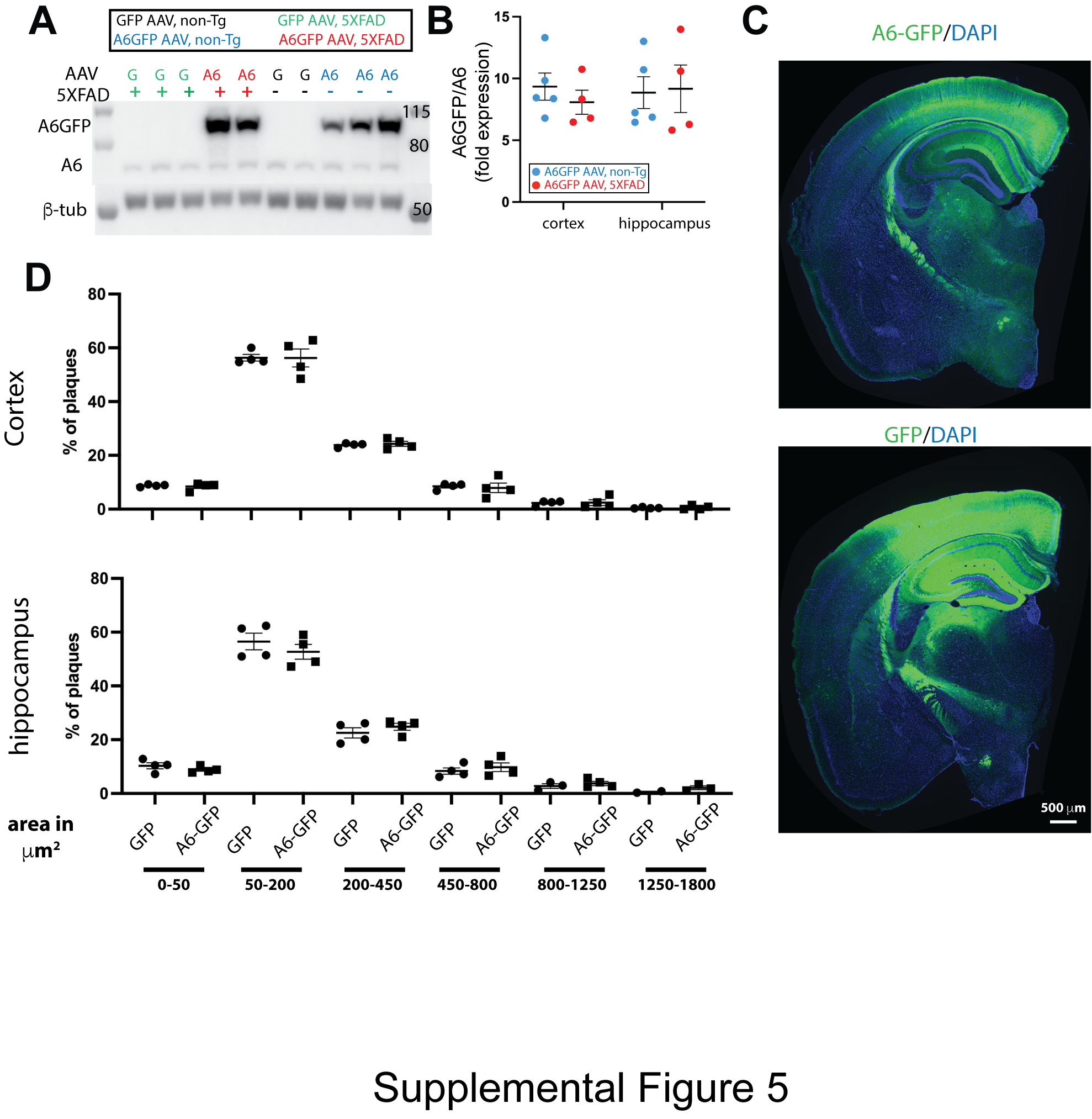

Supplement: Supplementary file 5 — Supplementary file5 (TIF 4400 kb) [file 401_2025_2888_MOESM5_ESM.tif]

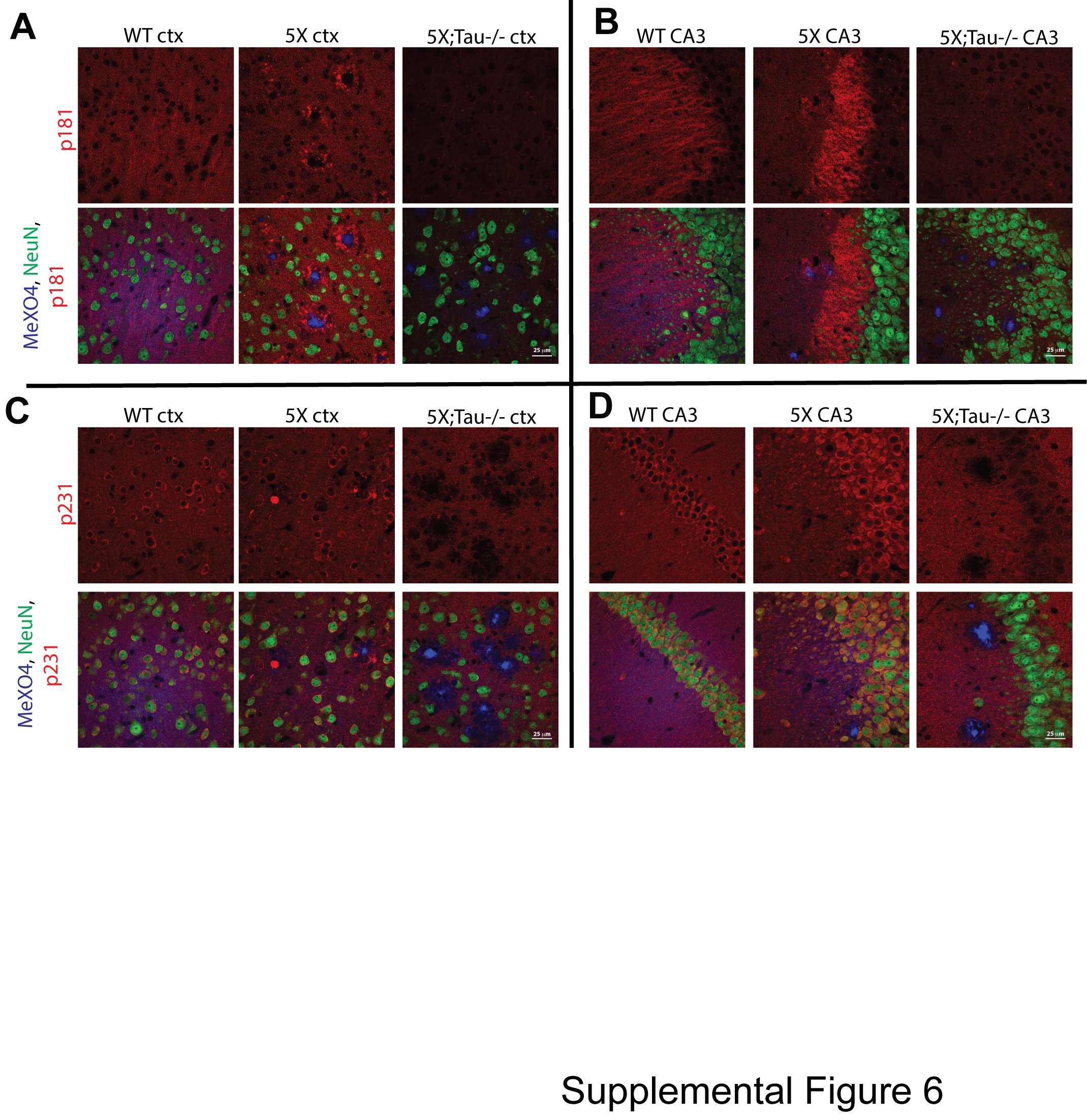

Supplement: Supplementary file 6 — Supplementary file6 (TIF 12372 kb) [file 401_2025_2888_MOESM6_ESM.tif]

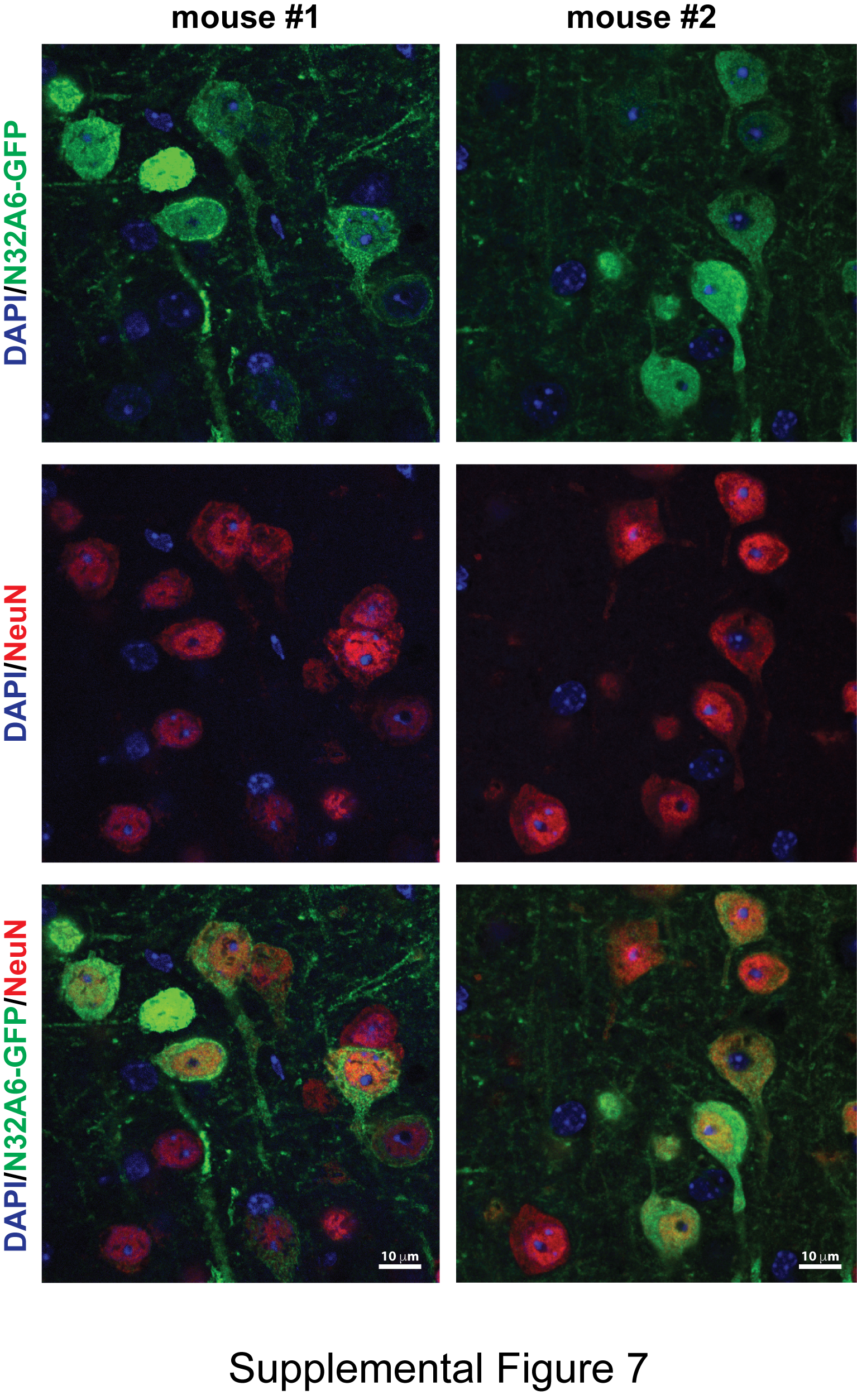

Supplement: Supplementary file 7 — Supplementary file7 (TIF 15094 kb) [file 401_2025_2888_MOESM7_ESM.tif]
