# Supplementary material for: Annexin A6 membrane repair protein protects against amyloid-induced dystrophic neurites and tau phosphorylation in Alzheimer’s disease model mice
Source: Acta Neuropathol. 2025 May 24;149(1):51. doi: 10.1007/s00401-025-02888-1 (PMC12103342; doi:10.1007/s00401-025-02888-1)
Supplement: Supplementary file 8 — Supplementary file8 (DOCX 19 kb) [file 401_2025_2888_MOESM8_ESM.docx]

**Supplemental Fig. 1:** **Validation of the GCaMP6s-ER/K-TdT calcium sensor.** (A) To confirm the calcium sensitivity of the sensor, SH-SY5Y cells were transfected with GCaMP6s-TDT, differentiated with retinoic acid, exposed to 90mM KCl to cause depolarization, and live-imaged by multiphoton microscopy over time. In the representative image in (A), cells that depolarize (green arrows in A, dashed lines in B) show a dramatic increase in GCaMPs:TDT ratio followed by a return to baseline. Some cells did not fire (red arrows in A, dashed lines in B), perhaps due to incomplete differentiation, and one dead/dying cell retained a high ratio throughout the experiment (yellow arow in A, dashed line in B). GCaMPs:TDT ratio in (A) is quantified over time in (B). C) Representative z-stack of 5XFAD mouse that was administered MethoxyX04, but did not receive an AAV syn-GCaMP6s-ER/K-TdT injection, confirming that high green and red channel fluorescence in plaque cores and puncta are due to autofluorescence rather than GCaMP6s and TdT fluorescence. D) The Z-stacks from which data in Figure 1C are obtained (left) and from which examples in Figure 1 B are cropped (right) demonstrating the range of ratios of GCaMP6s;TdTomato, along with examples of neurons with visible processes contacting plaques (arrows) or with no visible processes contacting plaques (arrowheads).

**Supplemental Fig. 2:** **Dystrophic neurites derive from both APP transgene and non-transgene expressing neurons.** Confocal images of 5XFAD mice (n=2, 8 month-old males) immunostained with DAPI (blue, nuclei and plaque dense cores), Karen antibody to human APP (green) and antibody to either BACE1 (red, first column) or Y188 antibody recognizing both mouse (m) and human (h) APP (red, second column). Not all dystrophic neurites containing BACE1 or m/hAPP (red) are positive for hAPP (green), indicating that transgene expression is not necessary for dystrophic neurite formation. Size bars, 10μm.

**Supplemental Fig. 3:** **Validation of the CFP-PLFAAR-YFP calpain sensor.** A) CFP-PLFAAR-YFP or CFP-GGGGS-YFP was transfected into HEK293 cells with or without co-transfection of FLAG-tagged calpain 1 large and small subunits and subjected to immunoblot analysis with anti-YFP and anti-FLAG antibodies. Cleavage of CFP-PLFAAR-YFP was only observed in the presence of large and small subunits of FLAG-tagged calpain 1. B, C) FRET between CFP and YFP was verified in fixed HEK293 cells and 5XFAD brain sections by demonstrating that after photobleaching the acceptor protein, YFP, fluorescence from the donor, CFP, increased, while FRET decreased. Arrowheads in B and C show a bleached HEK293 cell (B) and a bleached dystrophic neurite in a 5XFAD brain section (C). D) Examples of dystrophic neurite region of interest selection using CFP images. Individual panels showing CFP, YFP, and FRET channels, overlaid with Thiazine red (ThR) with bottom right panel showing the dystrophic neurites (dotted white line circles) discernible around plaques (asterisks) and cell bodies (arrows).

**Supplemental Fig. 4:** **Annexin A1 and A2 are present at high levels in vasculature and choroid plexus, but not in neurons.** A-C) Single cell RNA sequencing from the Human Protein Atlas and from brain and other tissues indicate divergent patterns of expression for annexins A1, A2 and A6. Annexin A1 is at low levels in the brain with higher levels in endothelial cells, and in the brain it is highest in certain subsets of excitatory neurons (A). Annexin A2 is also at very low levels in the brain, with higher expression in endothelium, but expression is confined to a subset of inhibitory neurons (B). Annexin A6 is found in all neuronal clusters in the brain at higher levels than annexins A1 and A2. Interestingly, the relative levels of annexins A6, A1 and A2 are similar in skeletal muscle and neurons, with annexin A6 being much higher than annexins A1 and A2, supporting the role of annexin A6 as the key mediator of membrane repair. Of note, in this brain single cell data set, vascular endothelia cells are not designated as a separate group, but are grouped together as cells expressing the endothelial markers CD34, PECAM1, SELE, and SLC2A1. VWF expressing cells are classified as “microglia”. (D,E) WT C57/B6 brain immunostained for annexins A1 or A2, NeuN, and DAPI indicates localization of annexins A1 and A2 around vasculature and choroid plexus, but none to little annexins A1 and A2 observed in the general neuronal population.

**Supplemental Fig. 5: Further characterization of 5XFAD mice expressing annexin A6-GFP or GFP after AAV transduction**. A) Immunoblot of brain homogenates from AAV A6-GFP and GFP-only expressing 5XFAD and non-transgenic (non-Tg) mice labeled with antibodies against A6 and β-tubulin (loading control). B) Quantification of immunoblot in B showing fold expression of A6-GFP over endogenous annexin A6 in cortex and hippocampus of 5XFAD and non-Tg mice expressing AAV A6-GFP. C) widefield view of hemibrain from A6-GFP (top) and GFP (bottom) expressing mice to illustrate anatomical distribution of expression after P0 injection. Size bar = 500μm. D) Quantification of the percent of total plaques in cortex (top) or hippocampus (bottom) falling into each size grouping. There is no difference in plaque size distribution between A6-GFP and GFP expressing mice.

**Supplemental Fig. 6:** **p-tau181 is found only in axonal compartments in wild type and 5XFAD brains, while p-tau231 is found in neuronal soma.** A,B) WT, 5XFAD and 5XFAD;Tau-/- mice were stained with antibodies to NeuN (green) and p-tau181 and MethoxyX04 to label plaques and imaged by confocal microscopy. P-tau181 is found in dystrophic neurites (A) in 5XFAD brains, and in mossy fibers (B) in wild type and 5XFAD, but shows no co-localization with NeuN in neuronal soma. C, D) WT, 5XFAD and 5XFAD;Tau-/- mice were stained with antibodies to NeuN (green) and p-tau231 and MethoxyX04 to label plaques and imaged by confocal microscopy. P-tau231 co-localizes wih NeuN in neuronal soma of cortex and hippocampus of both wild type and 5XFAD mice, and appears only occasionally in dystrophic neurites in 5XFAD brain. For both p-tau antibodies, staining is absent in 5XFAD;Tau-/- mice.

**Supplemental Fig. 7:** **Dominant negative annexin N32A6-GFP loses membrane localization in non-Tg mice.** A) Confocal images of cortical sections of non-Tg mice transduced on P0 with AAV8 expressing the dominant negative A6 truncation mutation, N32A6-GFP, driven by the neuron-specific synapsin promoter and immunostained for GFP (green), NeuN (red), DAPI (blue). Note the loss of N32A6-GFP localization from the plasma membrane toward the cytoplasm and nucleus, similar to changes observed in 5XFAD mice expressing N32A6-GFP (Fig. 7A).
